# Systematic review of memory assessment in virtual reality: evaluating convergent and divergent validity with traditional neuropsychological measures

**DOI:** 10.3389/fnhum.2024.1380575

**Published:** 2024-05-16

**Authors:** Valentina Mancuso, Eleonora Diletta Sarcinella, Francesca Bruni, Sara Arlati, Simona Gabriella Di Santo, Marco Cavallo, Pietro Cipresso, Elisa Pedroli

**Affiliations:** ^1^Faculty of Psychology, eCampus University, Novedrate, Italy; ^2^Department of Psychology, Research Center in Communication Psychology, Università Cattolica del Sacro Cuore, Milan, Italy; ^3^Institute of Intelligent Industrial Technologies and Systems for Advanced Manufacturing–STIIMA National Research Council of Italy–CNR, Milan, Italy; ^4^Department of Clinical and Behavioral Neurology, IRCCS Fondazione Santa Lucia, Rome, Italy; ^5^Department of Psychology, University of Turin, Turin, Italy; ^6^Department of Geriatrics and Cardiovascular Medicine, IRCCS Istituto Auxologico Italiano, Milan, Italy

**Keywords:** convergent validity, virtual reality, memory assessment, psychometric validation, ecological validity

## Abstract

**Introduction:**

The evaluation of memory is a crucial aspect of both cognitive research and clinical applications, as it offers valuable insights into an individual’s cognitive wellbeing and performance. Conventional neuropsychological assessments represent the established method for assessing different aspects of memory. Recent technological advancements, specifically in the field of virtual reality (VR), have introduced novel methods for evaluating memory.

**Objective:**

This systematic review aims to examine the current state of memory assessment using VR technologies, assessing the degree of convergence and divergence between VR-based memory assessments and conventional neuropsychological tests.

**Method:**

A systematic review of the literature was conducted searching PubMed, PsycINFO, Web of Science databases, leading to the incorporation of 24 studies. Studies were grouped according to the examined memory domain (episodic, prospective, spatial domain). Convergence and divergence validity were examined for each, and information on software and hardware features was collected.

**Results:**

This review demonstrates a notable alignment between VR-based memory assessments and conventional neuropsychological tests. Moreover, VR tasks have shown to exhibit associations with executive functions and overall cognitive performance. The inclusion of various ecological contexts, such as residential environments, commercial establishments, and simulated scenarios, serves to augment the ecological validity of memory evaluations conducted in VR.

**Discussion:**

The findings indicate that VR assessments demonstrate a functional perspective by effectively capturing the dynamic relationship between memory, executive functions, and overall cognitive performance. Nevertheless, it is imperative to acknowledge and tackle certain constraints that may hinder the widespread adoption and utilization of VR tasks. These limitations encompass factors such as restricted accessibility to VR tasks and the presence of heterogeneity in VR hardware and software. The dynamic and ever-changing nature of VR technology presents a range of potential avenues for future investigation and utilization in the domain of memory evaluation.

## 1 Introduction

The primary objective of cognitive assessment is to obtain a thorough understanding of an individual’s cognitive functioning and detect any possible impairment (Benton, 1994). Traditional assessment tools have played a crucial role in diagnosing and assessing the extent of cognitive impairments.

Due to the complex and interdependent nature of memory, researchers have set up a range of experimental and clinical assessments to evaluate memory and unravel its components.

The assessment of memory impairments and their implications for daily functioning holds substantial importance, considering the widespread occurrence of various illnesses that can compromise memory capabilities and subsequently affect everyday activities and overall quality of life, such as stroke and neurodegenerative pathologies.

Memory tasks commonly employed in clinical practice or experimental investigations on aging are structured to allow for precise control over task parameters and testing conditions. This intentional design facilitates the examination of the complex processes at play. However, it is important to note that traditional neuropsychological tasks are designed to elicit the individual’s optimal performance in ideal conditions, which may not effectively capture impairments in memory functioning that occur in everyday life situations. In fact, one possible critique of numerous clinical assessments of memory is their limited ecological validity (Pause et al., 2013). The notion of ecological validity pertains to the degree to which the experimental conditions faithfully replicate real-world settings (Tupper and Cicerone, 1990) or the extent to which the conclusions drawn from a study can be generalized to real-life contexts (Franzen and Wilhelm, 1996). Traditional evaluations demonstrate limited resemblance to ordinary, everyday memory situations (Parsons, 2011). In real-world scenarios, the act of memorization often occurs within environments, with high levels of noises and complexity. Furthermore, this cognitive process is frequently performed simultaneously with the completion of additional tasks, such as participating in dialog, ambulating, or engaging in problem-solving endeavors. The testing conditions in experimental and clinical settings differ significantly from the conditions described earlier. In these settings, participants usually perform their tasks in a quiet environment, receive clear instructions about the tasks, primarily work with one-dimensional material, and focus their attention solely on the task at hand. Multiple studies have provided evidence indicating that suboptimal performance on these assessments does not consistently align with compromised performance in practical contexts (Wilson, 1993; Sbordone and Long, 1996; Manchester et al., 2004; Bottari et al., 2009). The previously mentioned situation poses a challenge to the practicality of using traditional assessments as a means to make a diagnosis, intervene and offer appropriate support to individuals with cognitive impairments.

Moreover, the correlation between performance on conventional memory assessment tools (such as the California Verbal Learning Test–CVLT–or Wechsler Memory Scale-Revised–WMS-R) and indicators of daily functioning (such as self and informant memory diaries, patient and informant memory questionnaires, and clinical ratings) tends to be modest at most. Various studies (Goldstein and Scheerer, 1941; Sunderland et al., 1983; Kaitaro et al., 1995; Chaytor and Schmitter-Edgecombe, 2003) have examined this correlation and have reported only modest connections between the two domains.

The application of virtual reality (VR) offers a promising solution for evaluating memory in ecologically authentic and standardized environments. This cognitive assessment technology is of great interest due to its ability to simulate naturalistic environments, while maintaining safe and reproducible experimental conditions, under the complete control of the experimenter (Rizzo et al., 2008). According to Fuchs (2006), VR offers users an immersive experience within a dynamic virtual environment, allowing them to participate in cognitive and sensorimotor activities while interacting with virtual stimuli. One notable benefit of VR resides in its capacity to generate immersive environments that faithfully reproduce the sensory elements of the real world, including visual landscapes and audible dialogs. Moreover, VR can successfully integrate the cognitive and physical challenges that individuals face in their everyday activities. Therefore, one could argue that carefully designed VR tasks have the potential to more accurately represent real-world capabilities compared to traditional neuropsychological evaluations (Rizzo et al., 2008).

The utilization of various hardware platforms in the development of VR tasks for memory assessment studies demonstrates the wide array of immersive experiences that can be attained. The range of hardware options can be classified into three modalities: non-immersive, semi-immersive, and fully immersive (Takac et al., 2021). Each modality provides unique levels of immersion and interaction. Studies have shown that non-immersive VR setups, commonly implemented through the use of computers and tablets, generally entail user engagement with a two-dimensional screen. The participants interact with the virtual environment by utilizing conventional input devices, such as a joystick or a touchscreen pad. Semi-immersive VR experiences, as observed in research utilizing video projectors, provide a moderate degree of immersion. Video projectors are utilized to project a virtual environment onto a larger screen or physical space, thereby generating a heightened sense of immersion for participants, while simultaneously enabling them to maintain awareness of their physical surroundings. The utilization of head-mounted displays serves as a prime example of fully immersive VR setups, which provide the utmost level of immersion. Head-mounted displays fully obstruct the participant’s visual perception of the physical surroundings and substitute it with the simulated digital environment.

Besides the hardware used, the virtual environments can be distinguished into computer-generated or real-life scenarios captured using 360° technologies (Mancuso et al., 2024). In the realm of computer-generated virtual environments, a notable benefit lies in their substantial capacity for customization. Academic researchers possess the capacity to strategically construct scenarios that closely correspond to the specific goals of their memory evaluations. This characteristic holds significant value in situations where the objective is to establish controlled and standardized conditions for cognitive assessment. One additional advantage of computer-generated environments is their capacity for reproducibility. The replication of virtual scenarios is a straightforward process, thereby increasing the dependability of research outcomes. The attainment of consistency in the evaluation process among diverse participants or at different time intervals is attainable, thereby establishing a solid basis for rigorous scientific investigation.

Nonetheless, computer-generated virtual environments may exhibit a deficiency in terms of realism when juxtaposed with authentic real-life settings. Although advancements in technology have undoubtedly enhanced the feeling of being present and fully engaged in virtual environments, it is important to acknowledge that there are still constraints when it comes to accurately reproduce the depth and complexity of real-life experiences. The potential absence of realism in these virtual environments gives rise to inquiries regarding the ecological validity of memory assessments conducted within them.

On the other hand, the utilization of 360° technologies enables the depiction of real-life situations with an exceptionally elevated degree of authenticity (Borghesi et al., 2022; Mancuso et al., 2024). Individuals who are fully engaged in these settings frequently experience a sense of authentic presence, thereby augmenting the ecological validity of evaluations of memory. The inherent behavior and reactions of individuals are more prone to correspond with situations and stimuli encountered in actual life circumstances.

Moreover, the presence of stimulus variation in authentic situations can pose a greater challenge and elicit more effective engagement of memory functions compared to controlled computer-generated environments. The diverse array of sensory encounters can result in more genuine reactions, especially when evaluating memory within settings that closely mirror everyday experiences (Mancuso et al., 2023).

Nevertheless, actual situations present inherent difficulties. Computer-generated environments are more controllable and replicable compared to other types of environments. This is because computer-generated environments have the potential to introduce confounding variables and impede the standardization of assessment conditions. Moreover, the process of capturing authentic situations through the utilization of 360° technologies can impose a significant demand on resources, requiring the acquisition of specialized equipment, the employment of trained personnel, and occasionally encountering challenges related to the availability of suitable locations and logistical considerations.

Many studies have provided evidence of notable correlations between performance on VR tasks and traditional neuropsychological assessments that measure the same cognitive functions (Matheis et al., 2007; Armstrong et al., 2013; Lalonde et al., 2013; Nolin et al., 2016; Pedroli et al., 2020, 2022). This evidence confirmed that VR protocols effectively demonstrated construct validity. Moreover, prior studies have provided evidence that diverse VR assessments can successfully differentiate between two separate cohorts based on their task performance levels, for example, between older adults with dementia and non-cognitively impaired seniors (Rizzo et al., 1997; Rand et al., 2007; Cushman et al., 2008; Werner et al., 2009; Banville et al., 2010; Tarnanas et al., 2013; Allain et al., 2014; Zygouris et al., 2015). More interesting for the aim of the present systematic review, the evaluation of performance in VR tasks often involves comparing it to performance in equivalent real-world tasks (Waller et al., 2001; Cushman et al., 2008; Renison et al., 2012; Allain et al., 2014; Vallejo et al., 2017). For example, previous research has established notable associations between spatial learning, as evaluated using a virtual maze, and its real-world counterpart in the spatial domain (Waller et al., 2001). Furthermore, Cushman et al. (2008) have documented the existence of correlations between spatial navigation abilities as assessed in a virtual hospital lobby and those demonstrated in the corresponding real-world environment.

To date, a multitude of studies have made noteworthy contributions to the advancement of innovative VR tools that are specifically tailored for the assessment of memory functions. However, most of them have primarily focused on the validation of measures by using samples composed mainly by young individuals. Furthermore, a noteworthy concern is the dearth of research investigations that offer substantiation for the construct validity and convergent validity of assessments based on virtual reality. These studies must incorporate meticulous correlation analyses utilizing established neuropsychological tests.

The primary objective of this systematic review is to comprehensively evaluate and systematically synthesize the current body of literature related to the evaluation of memory utilizing VR technologies, examining the degree of convergence and divergence observed between VR-based memory assessments and conventional neuropsychological memory tests.

Secondly, our review examines the hardware and software characteristics of the applications for memory assessment and protocols for their use, posing particular attention on the use of computer-generated virtual environments vs. 360° video technologies.

## 2 Methods

Preferred reporting items for systematic reviews and meta-analysis (PRISMA) guidelines were followed (Page et al., 2021).

### 2.1 Search strategy

**FIGURE 1:**
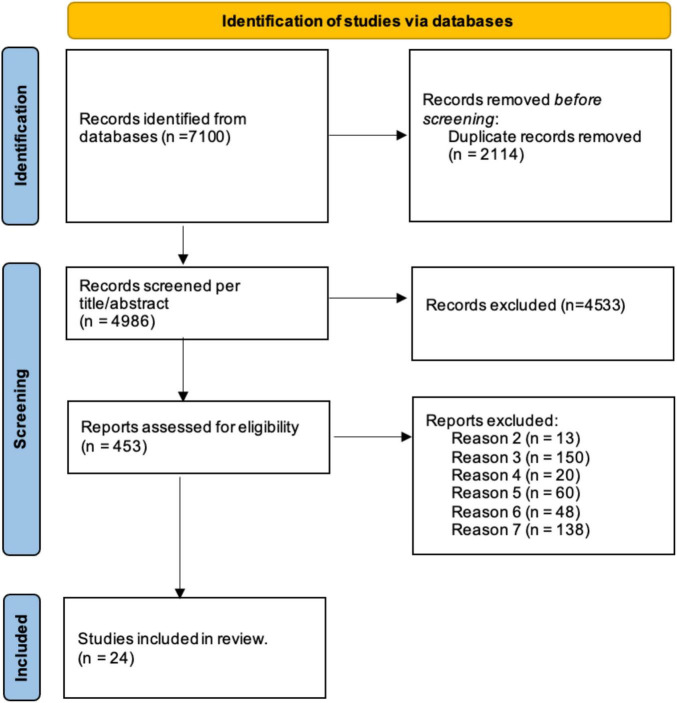
PRISMA flow diagram.

This section aims to provide a review of the studies that have assessed memory functions in virtual environments. Three high-quality databases (PubMed, PsycINFO, Web of Science) were used to perform the search on 4 August 2023. Keywords used for each separate string were (“memory”) AND (“assessment” OR “assess*” OR “evaluation” OR evalu* A) AND (“virtual reality” OR “VR” OR “virtual” OR “360-degree” OR “360°” OR “immersive” OR “panoramic”) and were searched through Title/Abstract, Abstract, Topic, respectively, for each database without time limit. Figure 1 shows the paper selection procedure and the number of selected/excluded articles.

### 2.2 Selection criteria

Studies that uniquely investigated the convergence and divergence validity of virtual reality-based memory assessment tasks with traditional neuropsychological measures have been included. In particular, we selected papers written in English (reason 1); experimental studies on human beings as reason 2 (we excluded reviews, meta-analysis, protocol articles, studies with animals, perspective); studies including participants over 60 years old (reason 3); use of virtual reality technologies (reason 4); assessment studies as reason 5 (we excluded usability, feasibility, rehabilitation and training studies); memory as cognitive domain evaluated (reason 6); studies with measures of convergency and divergency with traditional neuropsychological measures (reason 7).

## 3 Results

A total of 24 studies were identified and included in the present review.

**FIGURE 2:**
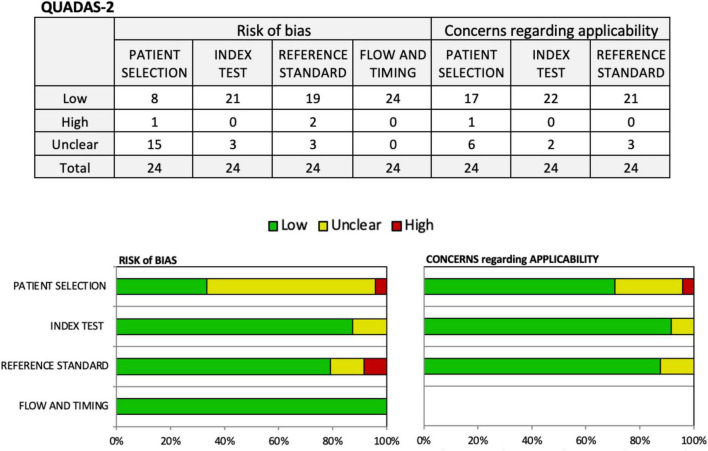
Assessment of risk of bias using Quadas 2 tool.

Table 1 presents a comprehensive overview of the VR assessment tools, providing a summary of their descriptions. Table 2 displays the convergence and divergence validity of these tools in relation to neuropsychological tests. Figure 2 shows the risk of bias assessed using the Quadas 2 tool (Whiting et al., 2011).

**TABLE 1:** Description of the Virtual assessment task, the hardware employed, and the virtual environment.

References	Memory	Virtual memory task	Hardware	Virtual environment
Arvind Pala et al., 2014	Episodic memory	HOMES TEST: Each participant visualized two versions (versions A and B) of a virtual apartment consisting of 40 objects: 20 objects that were specific to each version and 20 objects common to both versions. There were four categories (a bedroom, bathroom, kitchen, and a sitting room), therefore, each category contained 10 of these objects, five specific and five common to both versions. Participants first watched a target version of the apartment (called version A) which was presented twice and each participant had a free recall task immediately after each presentation. Immediately after the last recall task, participants visualized the other version of the apartment (version B) followed by only one immediate free recall task. After 10 min, a final yes/no recognition task was then administered to all participants.	The virtual environment was viewed using an F1+ overhead video projector on a 2 × 1.88 m large screen fixed onto the wall. Participants sat 2 m away from the display screen in a dark, silent room to visualize the VE projected in front of them.	Virtual apartment (a bedroom, bathroom, kitchen and sitting-room)
Sauzéon et al., 2016	Episodic memory	HOMES TEST: learning phase: participants first watched a target version of the apartment (version A). The target version was presented twice (trial 1 and 2) and each participant was asked to complete a free-recall task immediately after each presentation. Immediately after the second trial, participants visualized the other version of the apartment (version B) followed by only one immediate free-recall task. Recognition phase: After 10 min delay, a verbal yes/no recognition task was administered to participants as a final retrieval phase.	The virtual environment was viewed using a F1+ overhead video projector on a 2 × 1.88 m large screen fixed onto the wall. Participants sat 2 m away from the display screen in a dark, silent room to visualize the VE projected in front of them.	Virtual apartment (a bedroom, bathroom, kitchen and sitting-room)
Corriveau Lecavalier et al., 2020	Episodic memory	Virtual Shop (La boutique virtuelle): participants began the task in front of a cashier working behind a countertop and were presented with a list of 12 familiar virtual images of common items (e.g., belt, milk) that they were asked to memorize and then fetch in the store. During encoding, irrelevant conversations were presented via the head-gear in order to mimic a noisy environment. Following the presentation of the last item, the programme initiated a 20-s conversation between the cashier and the participant (e.g., Could you tell me the time, which is displayed on your right?) as a filled interference delay. At the end of the delay period, the cashier instructed the participant to fetch the items in the store he/she had previously seen. The participant could then walk freely in the room to find and select the items that were shown on the learning list. There were 24 items displayed in the shop: 12 target items and 12 distractors.	The virtual environment was in 3D and the immersion was produced by an Nvisor ST50 audio-visual headgear and by a Worldviz PPT-X studio tracking system that allowed the participant to rotate his/her head in a 360-degree view around the room, as well as look up and down, and interact and walk freely in the virtual environment. Participants used a hand remote control to select and retrieve items.	Virtual Shop (La boutique virtuelle)
Ouellet et al., 2018	Episodic memory	The Virtual Shop: The participant was first positioned in front of the countertop, with the shop shelves behind him/her. The experimenter explained to the participant that s/he would be presented with a list of items to memorize and that s/he would later have to “buy” these same items. The experimenter explained that the cashier of the shop will ask two questions the participant will have to answer and will then be instructed to start shopping. The pictures of 12 items were then presented on the flipping pages of a notepad situated on the countertop. When presentation was over, the cashier asked the participant a set of brief questions (e.g., “What is the weather like today?”) during 20 s as a way to empty the content of the participant’s working memory. The cashier then instructed the participant to walk into the store and fetch the objects that had been shown on the pad. The objects were located on shelves, inside refrigerators, hung on walls, or were placed on the floor. Twelve distractors that belonged to the same semantic category as the target items were placed in similar locations. Ambient verbal noise - a conversation between two customers - was presented dichotically through the HMD during the whole task.	HMD	Virtual Shop (La boutique virtuelle)
Pieri et al., 2022	Episodic memory	Encoding: the clinician moves around in the room and presents the target objects closely to the camera for 5 s; in the meanwhile, the participants must name the object showed. Free recall: The tasks simply require the participant to remember the 10 objects presented 10 min earlier in the Encoding Phase. Recognition: participants are asked to find and nominate all the ten objects previously showed in the encoding phase, located among other 17 non-target objects.	Oculus Go	Virtual medical office
Bruni et al., 2022	Episodic memory	OBRECO2: Encoding: Participants experience a household setting, such as a living room, in which they can with a first-person perspective which is the one of the experimenter. This one moves about the room highlighting the 15 target items for 3 s each and attaching a tag bearing the name “Marco” to each one. In the living room, there are also 15 other objects used as distractors. In this phase, participants are instructed to name all the targets. Free recall: After 15 min, hey are instructed to name as many objects from the encoding phase as they can. Recognition: Participants are instructed to explore the prior living room, discover and name the target objects among all of the previous things and an unknown set of 15 distractor objects.	Oculus Quest-2	Virtual living room
Kempe et al., 2015	Episodic memory	Supermarket test: participants had to perform two shopping tasks in counterbalanced order. In each, they saw a shopping list with 12 products. Each item was presented for 3.5 s on a computer screen, followed by a black screen for 0.750 s. Following the presentation, participants were asked to walk through the supermarket and to collect all products they remembered into a shopping basket. They were instructed to pick the products in the order they walk by them, which was not necessarily the order they appeared on the list.	Computer	Virtual supermarket
Zygouris et al., 2015	Episodic memory	Virtual supermarket (VSM): the patient is asked to navigate inside the virtual supermarket and buy the items displayed on a shopping list, appearing on the upper right corner of the screen. Upon purchasing all items of the list, s/he is required to locate the cash desk and proceed to pay. The payment screen is then displayed. The user must select the precise amount needed to pay for the items bought, using a selection of Euro bills and coins.	Tablet	Virtual supermarket
Eraslan Boz et al., 2020	Episodic memory	Virtual supermarket (VSM): a shopping list appears in the upper right corner of the screen during the VSM exercise. The person is expected to locate the items on this list, place them in the shopping cart, take them to the cashier desk and pay the correct amount for the purchases. The participant is asked to move the shopping cart and navigate inside the virtual supermarket by touching green footprints on the screen. It is an exercise designed for examining multiple cognitive domains such as visual and verbal memory, executive functions, attention, and spatial navigation.	Tablet	Virtual supermarket (VSM)
Limoncu et al., 2021	Episodic memory	The virtual supermarket test (VST): the participant is given a shopping list and is expected to place the items on this list in the shopping cart quickly and accurately and complete the shopping. Then, the user is expected to pay the correct amount for these products at the cashier desk. VST measures five variables: “Correct Items,” “Correct Quantities,” “Bought Unlisted,” “Correct Money,” and “Duration.”	Tablet	The virtual supermarket test (VST)
Turner et al., 2021	Episodic memory	Virtual Reality Functional Capacity Assessment Tool (VRFCAT): first, participants explore a kitchen to see what items they have for the recipe. Then, they use a bus schedule to find a bus that will take them to a grocery store. They find and purchase the necessary items at the store and use the schedule once again to find the bus that will take them home. Across the 4 scenarios, there is a total of 139 12 different tasks or “objectives.”	Tablet	Virtual kitchen, bus, grocery store
Plancher et al., 2010	Episodic memory	In the car accident scene, two cars crashed into one another, a horn was honked, and black smoke appeared. Buildings connected each specific area to the next. People, garbage containers, barriers, trees, billboards, and motion- less cars constituted some other elements of the town. Each specific area is composed of certain of these other elements. For example, in front of the town hall, there is a woman walking, a billboard, barriers, and trees. Free-recall test. After driving in the town, all participants underwent the same episodic memory tests. We asked for the free recall of verbal components in writing: the what, and where, when and details associated with the maximum of elements as possible. This test was limited to 5 min but in fact it was enough as no participant was stopped while still working. Recognition test: A recognition test was presented after the recall task. Participants had to choose which item they had seen in the town from among three different images. This test was com- posed of 10 questions concerning the elements and their locations in the town. Five questions were dedicated to the accident—for example, “Who did you see in front of the accident site?”—and five questions to other elements of the town, for example, “What color were the containers?.”	The environment was run on a PC laptop computer and explored with a virtual car using a real steering wheel, a gas pedal, and a brake pedal. It was projected with a video projector onto a screen 85 cm high and 110 cm wide. The participants were seated in a comfortable chair. The VE was projected 150 cm in front of them.	An urban environment based on photos of Paris
Gottlieb et al., 2022	Verbal memory	The VR-RAVLT places the participant in a virtual office with a virtual personal assistant (avatar) seated behind a desk. The avatar tells the participant a list of 15 places s/he needs to visit on the same day, and that s/he must recall as many as possible. The avatar informs the participant that as she will be leaving early for the day, she will repeat the list to ensure the participant remembers all the places (i.e., similar to the procedure employed in the GS-RAVLT). List B consists of 15 places that the participant would need to visit on the next day. Participant responses are recorded by a research assistant in a form similar to what is used in the GS-RAVLT.	HTC-Vive	Virtual office
Hogan et al., 2023	Prospective memory	Virtual Reality Prospective Memory Shopping Task (VRPMST): the ongoing task (12 errands) is completed by moving around the virtual shopping center and entering stores in a set order. The tasks are designed to mimic everyday activities completed at a shopping center. For time-based PM, participants are told that their doctor has instructed them to keep track of their heart rate; therefore, they need to check their heart rate (press H on the iPad) every 3 min. Participants can check their virtual watch (elapsed time appears for three seconds) by pressing the TIME button on the iPad. The VRPMST features two types of event-based PM tasks. Firstly, participants are told that they are keeping track of food expenditure. Therefore, after purchasing food items (4 items), they are required to collect a receipt. Secondly, participants are informed that they lost their glasses on their previous shopping trip. There- fore, they need to ask the center’s security guard every time they see one (4 times) whether they have found their glasses.	Dell M6700 note-book computer	Shopping center
Lecouvey et al., 2019	Prospective memory	After a familiarization phase, participants were informed that they would be immersed in this virtual city, this time to pick up a friend at the train station (i.e., at the end of this city), and they would have several intentions to fulfill along the way. Encoding: Participants were shown seven intentions on a laptop computer. For half of them, there was a strong link between the prospective cue and the retrospective component (Link-EB; e.g., buy a stamp booklet at the post-office), while for the other half, there was a weak link between them (NoLink-EB; e.g., buy a pair of glasses at the fountain). The remaining intention was a TB one repeated over time (i.e., take medication every 2 min). Then, to ensure that they were correctly encoded, a cued recall test was administered after the intentions had been presented. Storage: There was a 10-min interval between the encoding and retrieval of intentions, filled by the completion of questionnaires. Retrieval: They were reminded that they would have to pick up a friend at the train station and fulfill several intentions along the way. To do so, they had to stop the car at the appropriate time or place (i.e., prospective component) and tell the experimenter which action they had to perform (i.e., retrospective component).	The virtual environment was run on a PC laptop computer and projected onto a 180 cm × 240 cm widescreen. Participants could use two pedals (gas and brake) to drive the car through the city but they could not control the wheel.	Virtual town with various buildings, traffic lights, stores, trees, hoardings, parked cars, pedestrians crossing and corners
Tippett et al., 2009	Spatial memory	Two paths (A and B) were chosen with fixed start and end points. The VE task consisted of a set of three learning trials for Path A followed by one learning trial for Path B. For each trial, the participant first viewed the pertinent path through the city passively. Participants were instructed to pay attention to where various landmarks were located within the city and with respect to one another. After the path was completely viewed, participants were returned to the start position and were required to replicate the path to the best of their abilities. Path B served as an interference condition and was conducted using a separate path design in the same city environment to counteract perceptual priming effects. Following these trials were short-delay (5 min) and long-delay (20 min) recall trials, during which participants navigated Path A by memory alone, with no passive viewing component.	The virtual environment was presented to the participant using an LCD projector (Boxlight Corp). Interaction was achieved using a modified eight-way gameport joystick (PC Gamepad, Gravis, Inc.). The device allowed recording of both button-press responses as well as navigational control.	Sunnybrook City
Zhang et al., 2021	Spatial memory	Learning phase: Before the start of the entire experiment, participants were informed that the goal of the virtual environment task was to find and maintain a location, which would always be located at the same place during the following tests. They should always reach this same destination during the entire study. This destination is invisible, participants should find it according to the instructions and tasks during exploring the entire virtual environment in the following trials. Participants were provided with 9 learning trials. Each learning trial had a 90-s time limitation; if participants failed to reach the destination within 90 s, the trial terminated immediately and the next trial began. In the learning phase, participants could reach the destination by remembering the sequence of body turns of each Y-shaped intersection (egocentric strategy) or other strategies. To evaluate the navigation performance of each participant, the following parameters were calculated for the 9 learning trials: speed, distance error, rotation, percentage of successful people and percentage of successful trials. Probe phase: The probe trial shared the same maze structure as the learning phase but with no salient landmarks (i.e., all distal cues were removed).	Participants were comfortably seated in front of the computer screen and moved the joystick (Sony Dual- Shock 4) freely in a first-person view	Virtual maze
Morganti et al., 2013	Spatial memory	VR-Maze spatial task (VR-MT): participants faced the front of the computer screen and they were provided with one of the paper-and-pencil versions of five different complex mazes (PP-MT). In order to assess allocentric spatial knowledge in the PP-MT, they are requested to draw the path from start to exit trying to find the most efficient way between the two points. After each PP-MT was performed, in order to assess the allo- to egocentric translation of spatial knowledge, participants were asked to use the PP-MT in order to locate the exit point in the corresponding VR-MT. In the VR-MT exploration, a maze was considered as correctly performed if the participant was able to reach the exit point within the maximum time provided (10 min). Virtual Road Map task (VR-RMT): the participants faced the front of the computer screen with the paper version of the Money’s Road Map Test (PP-RMT) placed at the base of the screen. The PP-RMT consists of a stylized city map in which participants have to indicate on a 32-step dotted pathway the direction taken at each turn (left or right) in order to follow a designated route. Afterward, the participants were asked to use the PP-RMT to specifically navigate the VR-RMT by following the route indicated by the dotted line on the PP-RMT. The main objective of this task is to evaluate if the allo- to egocentric translation of spatial knowledge required for the navigation in the simulated virtual reality environment might differ from the one required from a mental imagery simulation of the same environment based on a sketched map.	All virtual reality tasks were administered on an Intel Core 2 Duo personal computer and presented on a 15″ desktop monitor. The participants were seated in a chair approximately 50 cm from the computer monitor and moved in VR using a narrow keyboard.	2 virtual mazes
Cushman et al., 2008	Spatial memory	Route learning: After completing the route demonstration, subjects were asked whether they had gone left, right, or straight at each of 10 choice points. Free recall: After the test trip around the route, subjects had 1 min to name as many objects or landmarks as they could recall. Self-orientation: Subjects were shown pictures of 10 different objects or locations from the test route, chosen to be distributed as two sequentially presented sets of five sites distributed at 45° intervals to the front and sides of the subject’s position, the subject’s back being toward an outside wall. Route drawing: Subjects used a mouse to indicate the location of the next choice point while viewing a scale map of the lobby on the video screen. Landmark recall: Subjects were asked to name only those objects or fixtures that were helpful in finding their way on the self-directed, second trip around the lobby. Photograph recognition: Ten photographs were presented singly on the screen, five from the test route and five from other locations in the Medical Center. Subjects identified whether each photograph was from the test route or not. Photograph location: Another set of 10 photographs from the test route was presented while subjects used a scale outline of the lobby with 10 locations marked by letters to indicate the location corresponding to the scene, scored as the number correct. Video location: Ten short video clips, taken from the subject’s view of the test route, were presented with three repetitions. After each display was completed, subjects drew an X on a blank map where the clip began and an arrow coming from the X showing the direction and extent of the depicted movement.	Laptot PC	Virtual hospital lobby
Serino et al., 2015	Spatial memory	A virtual room was created as test environment. It included two objects (namely, a plant and a stone) and an arrow drawn on the floor, which pointed to the North and represented the start of the navigation. Participants were instructed to memorize the position of the plant, that varied across three different trials. For the retrieval phase, two different tasks were developed. In the first task, participants were asked to indicate the position of the object on a real map, namely, a retrieval with spatial allocentric information independent of point of view. In the second task, participants were asked to enter an empty version of the same virtual room. The participants had to indicate the position of the plant, starting from the position of the other object, namely, a retrieval without any spatial allocentric information.	Participants were seated in front of a horizontally placed 15” monitor. The monitor screen was placed at a distance of 50 cm from the body plane. The virtual environments were rendered using a portable computer. The participants also had a gamepad which allowed them to explore and to interact with the environment.	Virtual room
Rekers and Finke, 2023	Spatial memory	VIENNA consists of one instruction trial, two practice trials, and 12 main trials. All trials show a first-person perspective of a character exploring virtual hallway environments. In addition, an allocentric map of the respective environment is displayed throughout each trial. Participants are required to mentally trace the character’s position and indicate the door that the character chose at the end of the trial. Importantly, this task design does not rely on episodic memory and does not require active exploration or navigation by the participant, thus homogenizing the available information to solve the task across participants.	Computer	Virtual hallway environments
Da Costa et al., 2022	Spatial memory	SOIVET Maze task: participants were required to navigate in a virtual maze using the route depicted on the original MRMT map as a reference. A green point marked the last correct turn on the map, in order to reduce working memory efforts. No topographical landmarks were provided. To navigate in the first-person perspective, participants were required to follow the route depicted on the map, but also update information from their body position at each turn on the maze. SOIVET Route task: participants entered the virtual reconstruction of the lobby of the Central Institute of the University of São Paulo Clinics Hospital. An avatar performed a route consisting of five specific locations inside the hospital lobby and its surroundings. Participants were required to follow the avatar in a first-person perspective. Subsequently, participants were required to repeat the same route alone, and to visit the five locations in the same order (SOIVET Route immediate). After a 20-min interval, the participants repeated the route one more time (SOIVET Route delayed).	Oculus Rift CV1 kit. Participants were able to navigate through the virtual environment using the touch controllers and their body position – turning the chair either right or left.	Virtual city
Mohammadi et al., 2018	Spatial memory	Virtual reality navigation task (VRNT): each of the virtual reality environments (virtual neighborhood and virtual maze) comprised a 3-D first-person view and a two-dimensional overhead view of the environment. First, the two- dimensional overhead view was shown to the subjects for 60 s, and then the 3-D first-person view was presented. Subjects were then instructed to find the specified goal (i.e., parking in the virtual neighborhood; the ball in the virtual maze), which had been marked on the 2-D overhead view. All subjects had three trials to familiarize themselves with the task and five trials for their assessment.	Computer and joystick	Virtual neighborhood and virtual maze
Lee et al., 2014	Spatial memory	The virtual radial arm maze (VRAM): participants were told that they were in a virtual room with six arms extending from a middle area. The virtual room had various colored objects and visual cues to indicate the relative directions, and the room remained unchanged throughout each trial. Although participants were instructed to find the three treasures as quickly as possible, no time limit was imposed. After discovering all three treasures, the trial ended, and participants returned to the center of the maze to begin the next trial. Five trials were conducted, and the intertrial interval was 10 s. The same configuration of rewarded arms was used for all participants. This test measured working memory errors by the number of times a subject reentered the same arm; reference memory errors were measured by the number of times a subject reentered the arms with no rewards. 22 Distance traveled, and time required to find all rewards during each trial were also recorded.	Desktop computer with a color monitor and a joystick	Virtual maze

**TABLE 2:** Description of the validation procedures: population employed, the VR task, neuropsychological measures administered, other measures, results of the VR task, and the correlation of this task with neuropsychological measures.

References	Population	VR task	Neuropsychological measure	Other measures	Results of the VR task	Correlations with neuropsychological tests
Arvind Pala et al., 2014	16 young students (age, M = 22.44, SD = 2.05); 15 healthy older adults (age, M = 66.53; SD = 3.43); 15 young moderate-to-severe TBI patients (age, M = 36.27, SD = 12.72).	HOMES TEST	Mini-mental state examination (MMSE); Mattis Dementia Rating Scale (MDRS); Trail Making Test (TMT); Stroop Task; Digit Symbol Substitution Test; Memory Self-evaluation Questionnaire – short form (QAM); California Verbal Learning Test (CVLT).	New technology (NT) experience; Simulator sickness questionnaire (SSQ)	Recall performances increased from trial 1 to trial 2. A *post hoc* comparison revealed significant group differences between young controls and older adults and between young controls and TBI patients, with higher recall performances in controls compared with older adults and with TBI patients, but not between older adults and patients. Overall, performances were better on the recognition task compared to the free recall task. *Post hoc* comparisons showed that older adults and TBI patients both performed less well compared with young controls. Also, healthy older adults and TBI patients had equally poor performances. Furthermore, there was a significant interaction, showing that the difference between performances on the recall and recognition tasks increased from young control to older adults and to TBI patients.	All of the neuropsychological indices were correlated with the HOMES learning and the HOMES corrected recognition.
Sauzéon et al., 2016	23 young adults (age, M = 22.20, SD = 1.68), 23 older adults (age, M = 73.60, SD = 9.10); 16 AD (age, M = 78.63, SD = 7.89)	HOMES TEST	MMSE; QAM-short form; mental rotation; Backward Corsi Block, Stroop test, CVLT.	NT, SSQ	Younger adults performed significantly better on the free-recall tasks than older adults, who in turn showed better recall performances than AD patients. The same ANOVA conducted with the retrieval tasks (free-recall trial 2 vs. recognition hits) used, as the within-subject variable showed, a main effect of group, and a main effect of retrieval tasks. The *post hoc* follow-up test revealed that poorer mean performances were recorded for older adults compared to younger adults, and these performances were even poorer in AD patients compared with both younger and older adults; overall, the performances were better on recognition than on free recall.	With regard to the relation between EF and HOMES true memory scores, the *r* value for the young group was not significant while that for other group conditions reached the significance. In summary, the HOMES true memory scores are mediated by both EF and EM scores, and an increased mediating effect of the EF score is obtained for old adults and the AD patients. In contrast, the HOMES false memory indices are only mediated by EM scores in such a manner that after controlling the EM score, the group effect is canceled. The self-reported memory complaints (QAM score) strongly correlated with the two HOMES memory indices indicating that low true memory and high false memory performances are associated with higher memory complaints in everyday life. Beside this, when the correlation analyses between QAM and HOMES indices are performed by group, they still remained significant only for young participants.
Corriveau Lecavalier et al., 2020	20 younger participants (age, M = 21.65, SD = 2.46); 57 older adults (age, M = 67.77, SD = 7.031)	Virtual Shop (La boutique virtuelle)	A validated free recall word list test			Performance on the VR task correlated with the immediate and delayed free recall scores of the traditional verbal memory task in both younger and older adults.
Ouellet et al., 2018	35 older adults with subjective cognitive decline (SCD) (age, M = 67.20, SD = 7.87).	Virtual Shop (La boutique virtuelle)	Multifactorial Memory Questionnaire (MMQ); Story Recall II; Stroop-Victoria test; MOCA.			Lower recall in the Virtual Shop task was associated with reporting more frequent difficulties related to shopping in daily life; the initiation time score of the Virtual Shop was also correlated with the MMQ-shopping complaint score; There was no association between time to complete the VR task and the MMQ-shopping complaint score. Importantly, performance on the Virtual Shop was not correlated with the global MMQ-ability score or with the MMQ-ability score that pulled out the 2 shopping items. This indicates that the relationship between the VR task and performance in daily life is specific to activities similar to those tested in the Virtual Shop. The scores for correct recall in the Virtual Shop task were positively correlated with traditional neuropsychological measure of episodic memory. Initiation time on the Virtual Shop was positively correlated with completion time for the first, second, and third plate of the Stroop-Victoria. The MMQ-shopping complaint score was positively correlated with completion time on the third plate. The low and high interference scores were not correlated with any of the VR scores.
Pieri et al., 2022	24 older adults (age, M = 70.4, SD = 8.5)	ObReco	MMSE; frontal assessment battery (FAB); picture recognition sub-test included in the Rivermead behavioral memory test (RBMT-III) Italian Version; Babcock Story Recall Test Italian Version.	Independent television com- mission-sense of presence inventory (ITC-SOPI), system usability scale (SUS)		The results indicate that for the Free Recall tasks, participants performed better after the 360° presentation than after the standard one (RBMT) in terms of accuracy percentages. For what concerns the Recognition indexes, the participants performed better in recognizing the objects after the standard presentation than after the 360° one.
Bruni et al., 2022	20 older adults (age, M = 68.2, SD = 5.45)	ObReco2	MMSE, FAB, the Babcock Story Recall Test (BSRT) Italian Version, the Rey Auditory Learning test (RAVLT), the Tower of London (ToL), attentive matrices (AM), test exploring Constructive Apraxia, TMT; Raven’s progressive matrices; Picture Recognition sub-test included in the RBMT-III Italian Version.	STAM, ITC SOPI, SUS.		The results indicate that for the free recall tasks, participants performed better after ObReco-2 than RBMT-III in terms of the number of targets correctly recalled although the difference is not statistically significant. Concerning the recognition indexes, participants recognized more objects after the standard presentation compared to the 360° one, and the observed difference is statistically significant. In the experimental group ObReco-2 scores correlate with AM and delayed RAVLT.
Kempe et al., 2015	20 young adults (age, M = 24.89, SD = 3.16); 23 older adults (age, M = 70.28, SD = 4.65)	Supermarket test	Digit span, grid test, digit complex span test, grid complex span, verbal simple span (VS), verbal complex span (VC), spatial simple span (SS), spatial complex span (SC), Stroop test.		Young adult’s memorized more items than older ones in all memory tasks domains. Time needed to fulfill the supermarket tasks was significantly lower for younger than for older participants. For older adults, as expected, the laboratory memory tasks correlated with each other. In the younger age group, just the verbal tasks significantly correlated with each other, but there was no connection between the two spatial difficulties or between the verbal and the spatial tasks.	Short term memory in the VR task correlated with VC for older adults and with SC for younger adults. The Stroop test did not show any connection with other tasks in the older group, but with EDS-M and EDS-STM in the younger group.
Zygouris et al., 2015	21 healthy older adults (age, M = 66.57, SD = 1.20), 11 single domain MCI (aMCI-SD) (age, M = 66.55, SD = 1.71), 23 multi domain amnestic MCI (aMCI-MD) (age, M = 72.13, SD = 1.50)	Virtual supermarket (VSM)	MMSE, RAVLT, Greek version of the “FAS” verbal fluency test, Rey-Osterrieth Complex Figure Test (ROCFT), RBMT, test of everyday attention (TEA) items 1, 4, and 6; TMT part B; functional rating scale for symptoms of dementia (FRSSD), functional cognitive assessment scale (FUCAS), clinical dementia rating (CDR); Greek dementia screening scale; Greek self-report questionnaire assessing early signs of cognitive decline.	Beck anxiety inventory (BAI), Beck depression inventory (BDI), geriatric depression scale (BDS), and the perceived stress scale (PSS).		The supermarket test duration significantly negatively correlated with MMSE, FUCAS, CDR, TRAIL B, TEA, RBMT, ROCFT, BAI. VSM correct types correlated with MMSE, TRAIL B, RBMT. VSM correct quantities correlated with MMSE FUCAS TRAILB RAVLT-learning abilities. VSM Bought Unlisted correlated with TRAIL B, TEA, RBMT, RAVLT.
Eraslan Boz et al., 2020	37 patients with aMCI (age, M = 70.41, SD = 7.297); 52 healthy controls (age, M = 67.56, SD = 6.044).	Virtual supermarket (VSM)	Oktem verbal memory processes test, Wechsler Memory Scale-Revised (WMS-R); visual reproduction subtest, Stroop test, digit span, figure copying test, clock drawing test, the Turkish version of revised MMSE, verbal fluency test (categorical and phonemic), Boston Naming Test, Wechsler Adult Intelligence Scale-III (WAIS-III) similarities subtest, Luria Alternan Sequences Test, Lawton Instrumental Activities of Daily Living (IADL).	BAI, GDS		The general cognitive status negatively correlated with correct quantities, bought unlisted, correct money and duration. Verbal memory and visual memory negatively correlated with all the measures of the VSM. Executive functions correlated with all measures except for correct money. Attention negatively correlated with bought unlisted, correct money and duration. Visuo-spatial construction negatively correlated with correct quantities, bought unlisted and correct money.
Limoncu et al., 2021	30 Healthy control (age, M = 67.27, SD = 7.79); 37 patients with small vessel disease cognitively normal (SVD-CN, age, M = 62.73, SD = 10.16); 32 patients with SVD with cognitive impairment (SVD-CI, age, M = 67.16, SD = 9.35).	The virtual supermarket test (VST)	MMSE, Oktem verbal memory processes test and Wechsler Memory Scale-Revised (WMS-R), visual reproduction subtest for visual and verbal memory; Stroop test, clock drawing test, phonemic fluency, Wechsler Adult Intelligence Scale–III (WAIS- III) similarities subtest, Luria Alternant Sequences Test; digit span; figure copying test for visuospatial function; Boston Naming Test and semantic fluency for language; IADL.	BAI, GDS.	A significant difference was found between SVD-CI and HC in terms of “Correct Types,” “Correct Money,” and “Duration.” In addition, there was a significant difference between SVD-CN and SVD-CI in terms of “Bought Unlisted” and “Duration.” However, there was no significant difference between SVD-CN and HC in VST variables.	There were negative moderate and strong correlations between “Duration” variable and general cognitive status and visuospatial functions on SVD-CI patients. In addition, a positive moderate correlation was found between “Correct Quantities” and general cognitive status on SVD-CI patients. The “Duration” variable was positively correlated to memory, executive function, and visuospatial functions on SVD-CN patients. The “Correct Money” variable was positively associated with executive function and negatively associated with visuospatial functions on SVD-CN patients. No significant correlation was found between VST variables and composite Z score of cognitive domains on HC.
Turner et al., 2021	14 PD patients (age, M = 65.5, SD = 7.38), 15 PD-MCI patients (age, M = 67.67, SD = 8.04)	Virtual Reality Functional Capacity Assessment Tool (VRFCAT)	MDRS, brief visuospatial memory test (BVMT-R, total learning and delayed recall), Hopkins verbal learning test (HVLT-R, total learning and delayed recall), judgment of line orientation (JOLO), neuropsychological assessment battery (NAB): numbers & letters A (time and errors), digits forward and digits backward, and naming subtest; Hayling sentence completion test (direct time, inhibition time, inhibition errors), controlled oral word association test (FAS and animals); TMT.	GDS, geriatric anxiety inventory (GAI)	For the overall sample, the average completion time for the VRFCAT-SL was 791 s (SD = 242) or about 13 min, with a minimum completion time of 504 s, and maximum completion time of 1,461 s.	T-score for VRFCAT-SL Time was positively associated with T-scores for NAB Numbers & Letters A Time, and BVMT-R Delayed Recall. The T-Score for VRFCAT-SL Errors was inversely correlated with Inhibition Errors (raw) on the Hayling, indicating greater errors on the VRFCAT-SL were associated with increased errors on the Haylling. T-score for VRFCAT-SL forced progressions was positively correlated with T-score for NAB Naming.
Plancher et al., 2010	78 healthy older adults (age, M = 63.73, SD = 10.74), 82 students (age, M = 21.47, SD = 2.99)	Car accident scene	MMSE; cognitive difficulties scale (CDS); TMT.		The *post-hoc* test exhibited no difference between young and older adults with intentional encoding, but the older adults scored higher in incidental encoding compared to the younger adults. In addition, there was a significant interaction on verbal where recalls. Indeed, *post hoc* Tukey’s tests indicated that the young participants performed better on verbal “where” recalls than the older participants on intentional encoding but also on incidental encoding. Moreover, there was an interaction on the visuospatial recalls, showing that the younger adults recalled more visuospatial information in intentional encoding compared to the older adults, although no difference between younger and older participants was observed in incidental encoding. An interaction was also observed when recalls, showing that the younger adults scored higher than the older adults in intentional encoding, but not in incidental encoding. Finally, it was observed an interaction in recognitions of the virtual town, indicating that the younger participants made more correct recognitions than the older adults in intentional, but not in incidental encoding. In other words, the performance of older adults was similar in both forms of encoding, but the performance of younger adults obviously increased in intentional encoding compared to incidental encoding. However, no effect of age and encoding was observed on the details memories.	The correct recalls and recognitions on the verbal episodic memory test were significantly correlated with the when recalls. However, other scores on the virtual town did not correlate with the scores of the verbal test, which in turn did not correlate with any scores on the neuropsychological tests. In addition, the TMT–A test score was significantly negatively correlated with the what score, the where score and the recognition score. By contrast, it did not correlate with the score on the verbal episodic memory test. Any of the scores on the VR and verbal episodic memory tests correlate with the TMT–B–A. Furthermore, the MMSE significantly correlated with score on visuo- spatial where, but the MMSE did not correlate with the verbal episodic memory test. Finally, the score on the CDS significantly correlated with the quantity of b0, and b1 and correct recognitions of the town. In contrast, CDS score did not correlate with score on the verbal episodic memory test.
Gottlieb et al., 2022	29 young adults (age, M = 24.1, SD = 2.9); 29 middle aged adults (age, M = 57.7, SD = 4.0); 20 Older adults (age, M = 71.8, SD = 5.1)	VR-RAVLT	RAVLT, MOCA, digit symbol test, digit span test, Verbal fluency.		Group effects were observed for the ACQUISITION and the RETENTION variables in the VR-RAVLT, respectively, with poorer scores for the older-adults group.	Statistically significantly correlations were found between the GS-RAVLT and the VR-RAVLT for two of the three outcome measures: ACQUISITION and RETENTION correlated with MOCA score, digit symbol test and verbal fluency. The digit span correlated only with VR-RAVLT acquisition. The retroactive interference did not correlate with any neuropsychological measures.
Hogan et al., 2023	12 individuals with stroke (age, M = 63.00, SD = 10.90), 12 controls (age, M = 55.33, SD = 9.95)	Virtual Reality Prospective Memory Shopping Task (VRPMST)	CAMPROMPT, lexical decision prospective memory task (LDPMT), TMT; HVLT-R, MoCA.	User−friendliness scale (UFS)	Both groups scored similarly on the ongoing task, indicating that they did not find the task too difficult. Controls performed significantly better on time-based PM compared to individuals with stroke (medium effect size). Additionally, controls monitored the time significantly more than individuals with stroke (large effect size). Controls scored higher than individuals with stroke on event-based PM; however, no significant difference was found. VRPMST monitoring significantly correlated with VRPMST time-based PM for both groups.	Time- and event-based VRPMST scores were significantly strongly correlated with both time- and event-based PM on the CAMPROMPT for the stroke sample. For controls, only event-based VRPMST significantly strongly correlated with both time- and event-based PM on the CAMPROMPT. Significant strong correlations were found between VRPMST time-based PM and LDPMT event- and time-based PM for the stroke sample but not for the control group. For individuals with stroke event-based PM on the VRPMST was significantly strongly correlated with the TMT, both HVLT- R scores, and MoCA, while time-based PM was significantly strongly correlated to the TMT, HVLT-R total recall score, and the MoCA. For controls VRPMST event- and time-based PM was significantly strongly correlated to TMT; however, only the HVLT-R percent retained score was significantly correlated with time-based PM.
Lecouvey et al., 2019	15 cognitively normal older individuals (age, M = 76.47, SD = 4.45); 17 patients with mild AD (age, M = 79.29, SD = 4.45).	Virtual city	MDRS, MMSE, RL- RI16 (retrospective episodic memory), picture-naming task, categorical and lexical fluency tests, Stroop test, TMT; Zoo Map test	BDI, State Trait Anxiety Inventory (STAI)	Cognitively normal older individuals needed lower number of trials to encode–and recalled more–Link-EB, NoLink-EB, and TB intentions when compared to patients with AD.	In AD patients, NoLink-EB intentions correlated with shifting tested by TMT part B and semantic memory. No correlation was significant with Link-EB intentions. Prospective component correlated with TMT part B and planning.
Tippett et al., 2009	26 control participants (age, mean = 69, SD = 7.7); 8 MCI patients (age, mean = 72, SD = 7)	Sunnybrook City task	ROCFT, TMT, digit span subtest (forward and backward), dementia rating scale, MMSE.	SSQ	Both participant groups were similarly successful in navigating Sunnybrook City to learn Paths A and B by memory. Both groups improved at a similar rate across trials, but overall MCI participants moved at reduced speed.	The VE navigation index showed significant correlation with the Trail Making Test (A error, and B error). The VE movement and memory indices did not show statistically significant correlations. The VE movement index also was significantly correlated with the Trail Making Test measures (A, A error, B error), whereas the VE memory index was correlated with measures of the Rey immediate recall and delayed recall.
Zhang et al., 2021	31 young adults (age, M = 22.68, SD = 3.07), 30 older adults (age, M = 67.30, SD = 5.10).	Virtual maze	Multifactorial Memory Questionnaire, the digit span (forward and backward), the block design (visuo-spatial abilities).		The main effect of learning trials were significant in speed, rotation, and distance error. The main effect of learning trials did not reach significance in the percentage of successful trials. Non-egocentric strategy users completed the virtual star maze task with more rotations and higher distance error. No significant effects were found in speed and percentage of successful trials.	For older adults, the WAIS block-design score was positively correlated with navigation speed and the percentage of successful learning trails, none of the other correlations were significant.
Morganti et al., 2013	26 early-stage Alzheimer’s patients (age, M = 80.96, SD = 6.3); 26 healthy participants (age, M = 77.23; SD = 5.25).	VR-Maze	MMSE, Corsi’s span; Supraspan test, TOL, TMT; Manikin’s test (body representations); benton line orientation test.		For the VR Maze spatial task the CG showed more correct performances than the AD group in the PP-MT and in the VR-MT.	The VR-MT results correlated with the mini-mental state examination, Corsi’s span, Corsi’s supra-span, Manikin Test and Trial Making Test. The VR-RMT results correlated with the mini-mental state examination and Corsi’s supra-span.
Cushman et al., 2008	35 young normal controls (age, M = 23.18, SD = 0.72), 26 older normal controls (age, M = 73.40, SD = 0.80), 12 MCI (age, M = 73.10, SD = 1.27), 14 early Alzheimer’ disease (age, M = 74.69, SD = 1.24).	Virtual hospital lobby task	Categorical name retrieval, Money Road Map test, line orientation, figural memory, verbal paired-associates test, MMSE.		In both the real-world and virtual environments, young subjects performed best, with successively lower scores in first the older normal group and MCI groups and then the Early AD group. There was a significant difference in performance between the two environments, with the virtual test yielding somewhat lower scores across all groups.	Stepwise multiple linear regression selected the virtual navigation and delayed verbal memory scores as the two best predictors of real-world navigation testing to results with an adjusted r2 of 0.82. The inclusion of MMS and judgment of line orientation scores enhanced the regression model to yield an adjusted r2 of 0.84. Thus, it seems that real-world testing might reflect the influence of factors well described by virtual testing along with factors well described by delayed verbal memory testing.
Serino et al., 2015	15 AD patients (age, M = 82.93, SD = 5.61), 15 aMCI patients (age, M = 77.53, SD = 5.52), and 15 cognitively healthy individuals (age, M = 73.87, SD = 7.38).	Virtual room task	Short story recall, MMSE, Milan overall dementia rating scale, Corsi block test, Money Road Map, Manikin’s test, judgment of line orientation.		For Task 1, there were no absolute significant differences between groups in the ability to retrieve spatial allocentric information independent of point of view. For task 2, *post hoc* comparisons indicated that AD patients performed more poorly when compared with the CG. This means that AD patients showed very weak abilities in retrieving the position of the object without allocentric spatial information.	A series of linear multiple regression analyses, including all participants, with the accuracy of spatial location for both tasks in each trials as the dependent variable, and general cognitive functioning (MMSE) and traditional Money Road Map, Corsi Block Test- Span, Corsi Block Test- Supraspan, Manikin’s Test, the judgment of line orientation as independent variables, were carried out. As concerns findings from the second tasks, results showed that these neuropsychological tests in combination with each other predict impairment in the ability to retrieve the position of the object without allocentric spatial information only in the Trial 2. However, findings revealed that there are only two significant predictors of performance in the third trial of the Task 2, namely, the scores on the Money Road Map and the scores on the Manikin’s Test. These two tests, indeed, evaluate, respectively, the ability in the spatial navigation, which requires the cognitive ability to correctly retrieve the position of the object in large environment, and the mental rotation ability, which is fundamental in the Trial 3, since it required a 180° spatial rotation to memorize the object.
Rekers and Finke, 2023	79 participants (age, mean = 67.8 years, SD = 8.89).	VIENNA task	ROCF, block tapping, visual memory span forward and backward, Wechsler Memory Scale-Revised Version, MMSE, Testbatterie zur Aufmerksamkeitsprüfung (TAP), Vandenberg’s mental rotation test (MRT), Five-point test, productivity, flexibility, and strategy (FPT), perspective taking test (PTSOT)	Complainer profile identification (CPI), the German translation of the Santa Barbara sense of direction scale (SBSOD); GDS	VIENNA scores in the sample were normally distributed in the upper half of the theoretically possible test score range of 0 to 24. The distribution of perspective rotation errors was slightly right-skewed while updating errors showed significant right skewness, with only 10 participants making more than one updating error. The median VIENNA administration time, including pretest and instructions, was 16 min. Furthermore, no outlier scoring below or above 2.5 standard deviations from mean VIENNA performance was observed.	VIENNA performance correlated significantly with the following tests: (i) large correlations with mini-mental state examination, visuospatial working memory, perspective taking, and visuoconstructive productivity; (ii) medium to large correlation with mental rotation; (iii) medium correlations with visuoconstruction and visuospatial short-term memory; (iv) and small to medium correlation with the executive function strategy application. VIENNA did not correlate significantly with episodic memory, operationalized by the percentage of recalled elements in the delayed free recall of the Rey-Osterrieth Complex Figure Test, cognitive flexibility or attention regarding processing speed, and selective attention. Correcting for participant age, all previously significant correlations, except for strategy application, remained significant, and effect sizes for correlations with working memory, productivity, and mental rotation decreased significantly. When correcting for average performance in neuropsychological tests, only the correlations with MMSE, perspective taking, and visuoconstructive productivity remained significant and showed medium-sized effects. Perspective rotation errors correlated significantly with the perspective taking test, but not with the mental rotation task.
Da Costa et al., 2022	20 cognitively healthy elderly (age, M = 70.21, SD = 5.28); 19 MCI (age, M = 72.53, SD = 6.50)	SOIVET Maze and SOIVET Route	ACE-R, Corsi block test, the Benton’s judgment of line orientation test (BJLO), TOL, MRMT.		The SOIVET Maze task was able to significantly differentiate groups while the MRMT showed no statistically significant difference. In the SOIVET Route task, the control group significantly outperformed the MCI group in the immediate phase as well as in the delayed phase.	The SOIVET Maze task revealed a significant correlation with the MRMT, Tower of London, BJLO test, and total ACE-R score. No significant correlation was found between the SOIVET Maze and Corsi Block Test forward or backward scores. Additionally, ACE-R- memory and ACE-R-visuo- spatial scores correlated with the SOIVET Maze. The SOIVET Route immediate also correlated with the MRMT and the ACE-R total score. In addition, the memory and visuospatial categories (*r* = 0.319, *p* = 0.045) from the ACE-R correlated with SOIVET Route immediate scores. SOIVET Route delayed scores did not correlate with any neuropsychological test, or with ACE-R total score and its categories.
Mohammadi et al., 2018	20 with miAD (age, M = 73.65, SD = 2.48), 30 with pure aMCIsd (age, M = 70.00, SD = 1.68), 30 with pure aMCImd (age, M = 70.07, SD = 1.67), and 30 cognitively normal controls (NC) (age, M = 69.87, SD = 1.43)	Virtual reality navigation task (VRNT)	MMSE; Boston Naming Test; RAVLT; R-OCFT.		Comparisons of the scores among the miAD, aMCIsd, aMCImd, and NC subjects across the five trials showed significant differences on the virtual neighborhood task. Further analysis showed that miAD patients performed significantly worse than the NC and aMCI subjects on the virtual neighborhood task. Similarly, the results indicated that the aMCImd subjects were significantly more impaired than aMCIsd and NC subjects, but they were less impaired than the miAD subjects. Comparing the performance of all groups across the five virtual maze trials showed significant differences in the response scores and response times. Tukey’s *post-hoc* test revealed that miAD subjects performed worse than the NC and aMCI groups on the virtual maze task. The response time analysis for all groups showed that miAD subjects required more time to complete the trials than the others. Also, there was a significant difference between aMCIsd and aMCImd subjects. A comparison of aMCI and NC subjects indicated that there were no significant differences with regard response scores or response time.	The results showed a strong positive correlation between neuropsychological and virtual neighborhood scores in all groups. A strong negative correlation was found between neuropsychological scores (RAVLT total scores, immediate recall, and delayed recall; R-OCFT immediate and delayed recall) and the mean response time for the virtual neighborhood task.
Lee et al., 2014	20 normal control (age, M = 70.8, SD = 5.2), 20 aMCI (age, M = 70.7, SD = 5.0), 20 AD (age, M = 72.4, SD = 5.6)	The virtual radial arm maze (VRAM)	Korean MMSE, CDR, simplified Rey figure test (SRFT); spatial span forward and backward.		Repeated measures ANOVAs revealed a significant main effect of number of trials on working and reference memory errors. All three groups committed fewer working and reference memory errors as the trials proceeded. Additionally, a significant effect of group was found on working and reference memory errors. According to the *post hoc* analysis, aMCI and NC participants committed a comparable number of working memory errors, but both groups committed fewer working memory errors than the AD subjects. aMCI subjects committed more reference memory errors than NC subjects and committed a similar number of reference memory errors to AD subjects. A significant main effect of trial was observed on distance traveled to find the rewards; hence, all three groups traveled shorter distances to find the rewards as the trials progressed. A significant main effect of group on distance traveled to find the rewards was also observed. Specifically, NC subjects found the rewards after traveling shorter distances than aMCI subjects and aMCI subjects found the rewards after traveling shorter distances than AD subjects. Finally, the data reflected a significant main effect of trial on time latency to find the rewards. All participants spent less time finding the rewards as the trials proceeded. Moreover, a significant group effect was found of latency, revealing that NC subjects found the rewards more quickly than aMCI subjects, whereas the time to find the rewards did not differ between aMCI and AD subjects.	The numbers of working and reference memory errors on the VRAM had significant linear correlations with each other and with scores on all neuropsychological tests except the Spatial Span Forward.

Through a thorough examination of the available literature, it emerges that VR tools that have been tested and proven to be reliable on older adults and have provided correlations with established neuropsychological assessments cover a wide array of memory areas such as episodic memory (Plancher et al., 2010; Arvind Pala et al., 2014; Kempe et al., 2015; Zygouris et al., 2015; Sauzéon et al., 2016; Ouellet et al., 2018; Corriveau Lecavalier et al., 2020; Eraslan Boz et al., 2020; Limoncu et al., 2021; Turner et al., 2021; Bruni et al., 2022; Pieri et al., 2022), verbal memory (Gottlieb et al., 2022), prospective memory (Lecouvey et al., 2019; Hogan et al., 2023), and spatial memory (Cushman et al., 2008; Tippett et al., 2009; Morganti et al., 2013; Lee et al., 2014; Serino et al., 2015; Mohammadi et al., 2018; Zhang et al., 2021; Da Costa et al., 2022; Rekers and Finke, 2023).

### 3.1 Research themes

#### 3.1.1 Virtual reality assessment tools for episodic memory

The model of episodic-like memory is a simplified representation of memory for episodes, which is characterized by three key conditions: the specific details of “what” occurred during an episode, the location or “where” it took place, and the temporal aspect of “when” it happened (Allen and Fortin, 2013).

The domain of memory assessment has experienced significant growth in recent years due to the increased accessibility of VR technologies. Nevertheless, it is important to highlight that a considerable proportion of these studies predominantly focused on comparatively younger demographics, despite the expected applicability of these methodologies for older individuals. Furthermore, it is worth noting that there is a noticeable deficiency in the existing body of literature, as several of these studies have neglected to include convergent validity measures, thereby restricting the thorough validation of these memory assessment tools that are based on VR. This specific section of the discussion aims to fill the existing gap in knowledge by shifting attention toward studies that have specifically focused on validating these tools among older populations. This overview aims to provide insights into the efforts made to determine the effectiveness and practicality of VR technology in evaluating episodic memory functions among older adults.

Two studies (Arvind Pala et al., 2014; Sauzéon et al., 2016) assessed episodic memory using the HOMES test: the participants were presented with a large display screen that depicted two versions (referred to as versions A and B) of a virtual apartment. Each version contained a total of 40 objects, with 20 objects unique to each version and 20 objects shared between both versions. There were four distinct categories, identified by the names of the rooms to which objects belong to: bedroom, bathroom, kitchen, plus an unspecified fourth category. Each of these categories consisted of ten objects, with five objects being specific to each category and five objects being common to both versions. The participants were initially exposed to a specific iteration of the apartment, referred to as version A. This iteration was shown to the participants on two separate occasions, and after each presentation, the participants were asked to engage in a free recall task. Following a duration of 10 min, a conclusive yes/no recognition task was subsequently administered to all participants. The first study compared older adults with traumatic brain injury (TBI) patients. Overall, there was an observed increase in performance levels from trial 1 to trial 2. A *post hoc* analysis was conducted, which indicated that there were no statistically significant differences observed between the two groups. In general, participants exhibited superior performance on the recognition task in comparison to the free recall task, albeit with suboptimal performance observed in both groups. In the study conducted by Sauzéon et al. (2016), a comparison was made between older adults and patients diagnosed with AD. The findings revealed that older adults exhibited superior recall performances in comparison to AD patients. The ANOVA analysis performed on the retrieval tasks (specifically, free-recall trial 2 versus recognition hits) revealed significant main effects for both group and retrieval tasks, as indicated by the within-subject variable. The results of the *post hoc* follow-up test indicated that older adults exhibited lower mean performances, and these performances were further diminished in individuals with AD when compared to other groups. In general, as expected the participants exhibited superior performance in the recognition task compared to the free recall task.

Another tool used to assess episodic memory is the Virtual Shop (La boutique virtuelle) (Ouellet et al., 2018; Corriveau Lecavalier et al., 2020). In this task, participants initiated the task by positioning themselves in proximity to a cashier situated behind a countertop. They were subsequently provided with a compilation of 12 virtual images depicting commonly encountered items (e.g., belt, milk), which they were instructed to commit to memory and subsequently retrieve within the store environment. During the process of encoding, extraneous dialogs were delivered through the use of headgear with the intention of replicating a disruptive auditory setting. After the conclusion of the previous item, the program implemented a 20-s conversation between the cashier and the participant. This conversation served as a filled interference delay, wherein the cashier asked the participant about the time displayed on their right, for example. Following the conclusion of the designated delay period, the cashier issued instructions to the participant, tasking them with retrieving the items within the store that they had previously observed. Subsequently, the participant was granted unrestricted mobility within the room in order to locate and choose the items that were previously presented on the learning list. The shop contained a total of 24 items, consisting of 12 target items and 12 distractors.

Obreco is another tool developed to assess episodic memory using 360° videos and images. In the first version (Pieri et al., 2022), participants wear a head-mounted display and adopt the viewpoint of the 360-degree camera. The clinician engages in movement within the room and brings the target objects in close proximity to the camera for a duration of 5 s. During this time, the participants are required to verbally identify the object being presented. Subsequently, the tasks solely necessitate the retention of the ten objects that were previously presented during the Encoding Phase, with a time interval of ten minutes. Subsequently, participants are instructed to locate and nominate all ten objects that were previously presented during the encoding phase, amidst a set of 17 additional non-target objects. In an upgraded version (Bruni et al., 2022), participants are exposed to a domestic environment, a living room, from the experimenter’s first-person perspective. They follow the perspective of the 360° video which selectively stops at the 15 designated objects for a duration of 3 s each, while simultaneously affixing a label inscribed with the appellation “Marco” to each item. In the living room, there are an additional 15 objects present, serving as distractors. During this phase, participants are provided with instructions to verbally label all of the targets. Following a period of 15 min, participants are given instructions to recall and identify as many objects as possible from the previous encoding phase. Subsequently, the participants are provided with instructions to thoroughly examine the previously observed living room environment, identify and label the specific target objects from the previously encountered items, as well as an additional set of 15 unfamiliar distractor objects.

One common task/environment frequently used to assess episodic memory is the supermarket task which typically requires memorizing a shopping list and then buying those products in the supermarket with additional different demands like picking the products in the order they walk by them (Kempe et al., 2015) or correctly select the precise amount needed to pay for the items bought (Zygouris et al., 2015; Eraslan Boz et al., 2020; Limoncu et al., 2021).

Similarly, in the Virtual Reality Functional Capacity Assessment Tool (VRFCAT) (Turner et al., 2021), a tool developed to measure functional capacity of subjective cognitive decline, participants engage in an exploratory activity within a kitchen environment to ascertain the available culinary resources for the recipe at hand. Subsequently, individuals employ a bus timetable to locate a suitable bus route that will transport them to a grocery store. The individuals locate and acquire the requisite items from the retail establishment, subsequently employing the aforementioned timetable to identify the bus that will facilitate their return to their place of residence.

With the same purpose but a different setting, Plancher et al. (2010) developed an episodic assessment tool set in a virtual urban environment based on Paris’ photos: At the site of a vehicular collision, two automobiles collided, resulting in the activation of a horn and the emission of black smoke. The interconnectedness of buildings facilitated seamless transitions between distinct areas. Various elements comprised the town, including individuals, waste receptacles, obstacles, vegetation, advertising displays, and stationary vehicles. Each specific area is comprised of a distinct combination of these various elements. Near the town hall, a pedestrian can be observed traversing the area, accompanied by a billboard, barricades, and a cluster of trees. Following their experience of driving in the town, all participants were subjected to identical episodic memory tests. Participants were instructed to engage in free recall of verbal components by providing written responses. Specifically, they were asked to recall the “what” (i.e., the content), “where” (i.e., the location), “when” (i.e., the time), and associated details of as many elements as they could remember. Following the completion of the recall task, a recognition test was administered. The participants were required to select the item they had observed in the town from a set of three distinct images. The examination consisted of a total of ten inquiries pertaining to the various elements and their respective positions within the municipality. The performance of older adults was similar in both forms of encoding, but the performance of younger adults obviously increased in intentional encoding compared to incidental encoding. However, no effect of age and encoding was observed on the details of memories.

Gottlieb et al. (2022) developed and validated a virtual reality-based Rey Auditory Verbal Learning Test (VR-RAVLT): VR-RAVLT immerses the participant within a simulated office environment, wherein a virtual personal assistant (represented as an avatar) is positioned behind a desk. The avatar provides the participant with a compilation of 15 locations that they are required to visit within a single day and subsequently challenges them to recall as many of these locations as they can. The avatar notifies the participant of her impending early departure and states her intention to reiterate the list in order to enhance the participant’s recall of all the locations, employing a procedure akin to that used in the standard RAVLT. List B comprises a total of fifteen locations that the participant is required to visit on the following day. The research assistant documents participant responses using a format akin to that employed in the GS-RAVLT. Group effects were observed for the *acquisition* and the *retention* variables in the VR-RAVLT, respectively, with poorer scores for the older-adults group.

#### 3.1.1.1 Convergence and divergence validity with traditional neuropsychological tests

The results of convergence and divergence of the virtual reality tests with neuropsychological tests are shown in Table 2. Overall, the HOMES (Arvind Pala et al., 2014; Sauzéon et al., 2016) scores correlated with all the neuropsychological measures; the Virtual Shop task correlated with the immediate and delayed free recall scores of the traditional verbal memory task in both younger and older adults (Corriveau Lecavalier et al., 2020) and traditional neuropsychological measure of episodic memory (Ouellet et al., 2018).

The OBRECO task (Bruni et al., 2022; Pieri et al., 2022) correlated with AM and RAVLT, i.e., executive functions and memory measures. The supermarket of Kempe et al. (2015) correlated with a verbal complex span; the supermarket of Zygouris et al. (2015) with most of the neuropsychological measures; the supermarket of Eraslan Boz et al. (2020) negatively correlated with general cognitive status, verbal and visual memory and visuo-spatial construction but positively with executive functions. The supermarket of Limoncu et al. (2021) correlated with memory, executive functions and visuo-spatial functions. The VRFCAT (Turner et al., 2021) was correlated with neuropsychological assessment battery (NAB) and delayed recall. The VR-RAVLT (Gottlieb et al., 2022) positively correlated with the standard RAVLT.

#### 3.1.2 Virtual reality assessment tools for prospective memory

Regarding prospective memory (PM), two virtual tools have been developed: the Virtual Reality Prospective Memory Shopping Task (VRPMST) (Hogan et al., 2023), and a ride in a virtual car (Lecouvey et al., 2019). Prospective memory refers to the cognitive ability to remember and successfully execute intended actions or tasks at a future point in time. The differentiation between time-based (TB) and event-based (EB) intentions is commonly made based on the characteristics of the stimulus that initiates the retrieval process.

The Virtual Reality Prospective Memory Shopping Task (VRPMST) (Hogan et al., 2023) involves the completion of a task consisting of 12 errands within a virtual shopping center. Participants navigate through the virtual environment and enter stores in a predetermined sequence. The tasks have been designed to replicate the routine activities typically carried out within a shopping center setting. In the context of time-based project management, individuals are informed by their healthcare provider to monitor their heart rate. Consequently, they are required to assess their heart rate at regular intervals of 3 min by pressing the designated key on the iPad. Participants can verify the elapsed time on their virtual watch for a brief duration of three seconds by using the TIME button on the iPad. The VRPMST encompasses two distinct categories of event-driven project management tasks. Initially, participants are informed that they will be monitoring their food expenses. Consequently, upon the acquisition of food items, specifically four items, it is necessary for individuals to obtain a receipt. Furthermore, the participants are provided with information regarding the loss of their glasses during their previous shopping excursion. Consequently, it is necessary for them to inquire with the security personnel at the center on each occasion (a total of four times) regarding the potential retrieval of their glasses. This test has been validated in two groups: individuals with stroke and controls. Both groups scored similarly on the ongoing task, indicating that they did not find the task too difficult. Controls performed significantly better on time-based PM compared to individuals with stroke. Additionally, controls monitored the time significantly more than individuals with stroke (large effect size). Controls scored higher than individuals with stroke on event-based PM; however, no significant difference was found. VRPMST monitoring significantly correlated with VRPMST time-based PM for both groups.

In the study of Lecouvey et al. (2019), following a period of familiarization, the participants were notified that they would be immersed in a virtual city with the objective of retrieving a companion from the train station, located at the conclusion of said city. Additionally, they were tasked with fulfilling various intentions throughout their journey. The participants were presented with a set of seven intentions displayed on a laptop computer. In 50% of the cases, a significant association was observed between the anticipated cue and the subsequent retrospective component (referred to as Link-EB). Examples of Link-EB scenarios include purchasing a stamp booklet at the post-office. Conversely, in the remaining 50% of cases, a weak association was found between the anticipated cue and the retrospective component (referred to as NoLink-EB). An example of a NoLink-EB scenario is buying a pair of glasses at the fountain. The persistent intention involved the regular administration of medication for tuberculosis, with a frequency of once every two minutes. Subsequently, to verify the accuracy of the encoding process, a cued recall assessment was conducted after the presentation of the intentions. A 10-min interval was observed between the encoding and retrieval of intentions, during which participants completed questionnaires. The individuals were reminded of their obligation to collect a companion from the train station and accomplish various objectives during their journey. In order to accomplish this, participants were required to bring the vehicle to a halt at the designated moment or location (referred to as the prospective component) and subsequently inform the experimenter of the specific action they were instructed to execute (known as the retrospective component). This test has been administered in patients with mild AD and controls: cognitively normal older individuals needed a lower number of trials to encode–and recalled more–Link-EB, NoLink-EB, and TB intentions when compared to patients with AD.

#### 3.1.2.1 Convergence and divergence validity with traditional neuropsychological tests

The results of the first study indicate that VR tasks are successful in capturing and reflecting the PM abilities that are evaluated by conventional measures. Moreover, the study unveiled significant correlations between time-based prospective memory tasks conducted in VRPMST and the corresponding time- and event-based PM tasks conducted in laboratory-based dual-task paradigms. Furthermore, a significant and strong association was observed between both time and event-based PM as evaluated using VRPMST and the outcomes obtained from the Trail Making Test (TMT), the Hopkins Verbal Learning Test-Revised (HVLT-R), and the Montreal Cognitive Assessment (MoCA).

In the second study, the NoLink-EB intentions (NoLink Event-Based) study revealed correlations between cognitive functions, specifically shifting as assessed by the Trail Making Test Part B, and semantic memory. Nevertheless, the study did not uncover any noteworthy associations with regards to Link-EB intentions (Link Event-Based).

In addition, it is worth noting that the potential aspect of PM exhibited a positive correlation with the TMT-B, a measure commonly used to assess cognitive flexibility and executive functioning, as well as with planning abilities.

#### 3.1.3 Virtual reality assessment tools for spatial memory

Among all the studies investigating spatial memory in VR, only 9 of these compare the performance of older adults with classical neuropsychological tests:

Tippett et al., 2009, chose two routes, Path A and Path B, with predetermined starting and ending points. Path A had three learning trials and Path B was one in the VE task. Each trial began with passive observation of the city path. Participants were instructed to focus on the city’s landmarks’ spatial positioning and relative positions. After thoroughly observing the path, participants were instructed to return to the starting point and reproduce it as best they could. Path B was used as an interference condition with a different path design in the same urban setting to reduce perceptual priming effects. After these experimental trials, participants performed recall trials with 5- and 20-min delays. They navigated Path A by memory without passive viewing during these trials.

In Zhang et al., 2021, the virtual environment task was to find and maintain a fixed position for nine learning trials. Participants must arrive at the same place throughout the study. In subsequent trials, participants must use instructions and tasks to find the location while navigating the virtual environment. The trial ended immediately if participants did not reach the destination within 90 s, and the next trial began. Participants could use the egocentric strategy—remembering sequential body turns at each Y-shaped intersection—or other strategies to reach the target location during the acquisition phase. Each participant’s navigational proficiency was assessed using several metrics for the 9 learning trials. This included speed, distance error, rotation, percentage of successful individuals, and percentage of successful trials. The probe trial maze structure was similar to the learning phase, but without landmarks. All distal cues were intentionally removed.

Morganti et al. (2013) created two spatial assessment tasks with no landmarks and uniform building textures. Participants were shown one of five complex paper-and-pencil mazes (PP-MT) on a computer screen in the VR-Maze spatial task (VR-MT). The PP-MT tests allocentric spatial knowledge by having participants draw the trajectory from the starting point to the exit and find the best route. After completing PP-MT, participants were instructed to use it to find the exit point in the VR-MT. This assessed the transfer of allocentric to egocentric spatial knowledge. To begin VR-MT study, participants were shown the correspondence between the initial positions on the physical paper and the virtual representation of each maze. Participants had 10 min to reach the exit point to complete a maze.

Participants faced the computer screen with the Money’s Road Map Test (PP-RMT) at the bottom in the VR-RMT task. In order to navigate a predetermined route on a stylized urban map, the PP-RMT requires participants to indicate their left or right direction at each turn using 32 dotted steps. Since the dotted pathway has a non-linear trajectory that goes both away from and toward the person, the solutions require egocentric mental rotation. The individual cannot manipulate the map or make any head or body movements to find the correct answer. Participants were then instructed to use the PP-RMT to navigate the VR-RMT by following the dotted line on the PP-RMT. The purpose of this task is to compare the process of converting spatial knowledge from an allocentric to an egocentric frame of reference for navigating a simulated virtual reality environment to mentally simulating the same environment using a sketched map.

Cushman et al. (2008) created eight tasks. Participants saw the route on a computer screen during route learning. The second presentation video playback stopped at ten decision points. After the test trip along the designated route, participants had 1 min to verbally identify and recall as many objects or landmarks as possible. In the Self-orientation phase, participants saw 10 images of test route objects and locations. These images were carefully selected and presented sequentially in two sets of five. The subject faced an outside wall and the locations were strategically placed at 45° intervals. Participants used a computer mouse to mark the next decision point while viewing a scaled lobby on the video display during route drawing. In the Landmark experiment, participants were instructed to recall only those objects or fixtures that helped them navigate their second lobby traversal. Ten photos were displayed on the screen for photograph recognition. Five photos were chosen from the test route and five from the Medical Center. The subjects determined whether each photograph was from the test route. Participants were shown 10 more test route photos during the Photograph location task. They received a scale lobby outline with 10 lettered locations. Participants had to indicate the scene outline location for each scene, and their performance was measured by correct responses. The previous task, the video location task, showed ten brief video clips of the subject navigating the test route three times. After each visual presentation, participants marked an X on an empty map to indicate the clip’s start. They also drew an arrow from the X to show movement direction and magnitude. Correct responses were based on X placement and arrow direction.

In Serino et al. (2015) created a controlled simulated room for experimentation. Two entities—a botanical specimen and a mineral specimen—were displayed with an arrow on the ground. This north-facing arrow symbolized the start of navigation. Three trials were given to participants to memorize the plant’s positions. Two retrieval tasks were created. Participants first had to locate the object on an authentic map. They were asked to recall spatial allocentric information independent of their perspective. The second task required participants to access an empty virtual environment. In a retrieval task without spatial allocentric information, participants had to place the plant relative to another object.

Rekers and Finke, 2023 tested the feasibility of VIENNA, which comprises a single instruction trial, followed by two practice trials, and finally, 12 main trials. The trials conducted exhibit a visual representation from the viewpoint of a protagonist engaging in the exploration of virtual hallway environments. Furthermore, a representation of the environment is presented in the form of an allocentric map during every trial. Participants are instructed to engage in mental tracing of the character’s position and subsequently indicate the door that the character selected at the conclusion of the trial. Significantly, the design of this task does not depend on episodic memory and does not necessitate active exploration or navigation by the participant. As a result, the information available to solve the task is made uniform across all participants.

Da Costa et al., 2022 created the SOIVET Maze and Route Tasks. Participants navigated a virtual maze using the original MRMT map in the first one. A green indicator on the map indicates the most recent accurate direction change to reduce cognitive load. No topographical landmarks were located. Participants were told to follow the map’s route to navigate first-person. They also had to update their spatial awareness by using their body position at each maze junction. The second task, SOIVET Route task, involved participants accessing the virtual lobby reconstruction at the Central Institute of the University of São Paulo Clinics Hospital. A virtual avatar performed a predetermined sequence of five locations in the hospital lobby and surrounding area. Participants were told to track the avatar in first-person. After that, participants were told to independently travel the same route and visit the five designated locations in the SOIVET Route immediate order. After a 20-min break, participants repeated the route with an SOIVET Route delay.

The virtual reality navigation task (VRNT) by Mohammadi et al. (2018) used two environments, the virtual neighborhood and virtual maze, with three-dimensional first-person and two-dimensional overhead views. Participants first saw a two-dimensional aerial view for 60 s, then a three-dimensional first-person view. After seeing the two-dimensional aerial perspective, participants were told to find the goal (e.g., parking in the simulated neighborhood, the ball in the virtual maze). Each participant had three practice trials to learn the task, then five assessment trials.

Lee et al. (2014) created the virtual radial arm maze (VRAM), in which participants were told they were in a simulated environment with a central region and six appendages. The virtual room had colorful objects and visual cues for directions. The room was unchanged throughout each trial. Although instructed to find the three treasures quickly, no time limit was set. After finding all three treasures, the trial ended, and the participants reconvened at the maze’s center for the next trial. Five trials were run with 10-s intertrial intervals. All participants received the same rewarded arms. Working memory errors were measured by reentry into the same arm, while reference memory errors were measured by reentry into arms without rewards. The distance and time taken to find all rewards in each trial were also recorded.

#### 3.1.3.1 Convergence and divergence validity with traditional neuropsychological tests

The research conducted by Mohammadi et al. (2018) revealed noteworthy associations between their spatial memory task and established memory assessments, such as Rey immediate recall and delayed recall. Significant correlations were observed between the VR-MT and VR-MTM tests and Corsi’s supra-span task, which is a commonly employed assessment tool for evaluating visuospatial memory and navigation abilities.

There was a notable association observed between the Vienna task and visuospatial short-term memory. Nevertheless, the present task failed to exhibit a substantial correlation with episodic memory, as assessed by the proportion of elements recalled during the delayed free recall phase of the Rey-Osterrieth Complex Figure Test (ROCFT).

In contrast, the study conducted by Mohammadi et al. (2018) found a correlation between the ROCFT and simplified Rey figure test (SRFT) with the virtual neighborhood task. Similarly, Lee et al. (2014) observed a correlation between ROCFT and SRFT with the virtual radial arm maze (VRAM). Additionally, Tippett et al. (2009) proposed a task that also exhibited a correlation with ROCFT and SRFT. These observations imply that the conventional visuospatial memory tests exhibit similar characteristics to their virtual reality counterparts, thus indicating their concurrent validity in evaluating spatial memory.

Just like tasks that evaluate episodic memory, a number of spatial memory assessments also exhibited associations with executive functions. In particular, Tippett et al. (2009) and Morganti et al. (2013) reported significant correlations between the Trail Making Test (TMT) and the variables under investigation. On the other hand, Rekers and Finke (2023) identified significant associations between visuospatial working memory, perspective-taking, and mental rotation. Furthermore, Da Costa et al. (2022) have documented correlations between the Tower of London task and virtual spatial memory assessments, suggesting that the latter may encompass elements of executive functioning.

Multiple studies have also demonstrated associations between evaluations of virtual spatial memory and overall cognitive functioning. In their study, Morganti et al. (2013) established associations between the mini-mental state examination (MMSE) and both VR-MT and VR-RMT. The study conducted by Rekers and Finke (2023) revealed a significant correlation between the VIENNA task and the mini-mental state examination (MMSE). Moreover, the study conducted by Lee et al. (2014) revealed significant associations between Virtual Reality Assessment Measure (VRAM) scores and MMSE scores. In contrast, Da Costa et al. (2022) identified associations between the Addenbrooke’s Cognitive Examination (ACE) and the SOIVET Maze task, underscoring the capacity of virtual spatial memory evaluations to serve as indicators of broader cognitive performance.

### 3.2 Populations

The demographic groups primarily subjected to examination through the utilization of VR tools for the purpose of memory assessment primarily consist of healthy older adults (Cushman et al., 2008; Tippett et al., 2009; Plancher et al., 2010; Morganti et al., 2013; Arvind Pala et al., 2014; Lee et al., 2014; Kempe et al., 2015; Serino et al., 2015; Zygouris et al., 2015; Sauzéon et al., 2016; Mohammadi et al., 2018; Lecouvey et al., 2019; Corriveau Lecavalier et al., 2020; Eraslan Boz et al., 2020; Limoncu et al., 2021; Zhang et al., 2021; Bruni et al., 2022; Da Costa et al., 2022; Gottlieb et al., 2022; Pieri et al., 2022; Rekers and Finke, 2023), older adults with subjective cognitive decline (Ouellet et al., 2018), individuals diagnosed with Mild Cognitive Impairment (MCI) (Cushman et al., 2008; Tippett et al., 2009; Serino et al., 2015); MCI-single domain (SD) (Lee et al., 2014; Serino et al., 2015; Zygouris et al., 2015; Mohammadi et al., 2018; Eraslan Boz et al., 2020), MCI-multiple domain (MD) (Zygouris et al., 2015; Mohammadi et al., 2018), patients with Parkinson’s disease (PD) (Turner et al., 2021), and PD-MCI (Turner et al., 2021), cognitively normal individuals with small vessel disease (SVD) (Limoncu et al., 2021), individuals with SVD with cognitive impairment (Limoncu et al., 2021); patients with stroke (Hogan et al., 2023), patients with AD (Cushman et al., 2008; Morganti et al., 2013; Lee et al., 2014; Serino et al., 2015; Sauzéon et al., 2016; Mohammadi et al., 2018; Lecouvey et al., 2019). See Table 3 for descriptions of the samples.

**TABLE 3:** Descriptions of the populations.

Population	Numerosity
Healthy older adults	655
TBI	15
Subjective cognitive decline	35
AD	91
Mild AD	37
MCI	39
aMCI-SD	113
aMCI-MD	23
Small vessel disease without cognitive impairment	37
Small vessel disease with cognitive impairment	32
PD	14
PD-MCI	15
Stroke	12
Young adults (control group)	285

### 3.3 Hardware

Among the studies under review, five employed video projectors as the primary tool for conducting virtual reality tasks (Tippett et al., 2009; Plancher et al., 2010; Arvind Pala et al., 2014; Sauzéon et al., 2016; Lecouvey et al., 2019). Six studies utilized HMDs as their selected hardware (Ouellet et al., 2018; Corriveau Lecavalier et al., 2020; Bruni et al., 2022; Da Costa et al., 2022; Gottlieb et al., 2022; Pieri et al., 2022).

Nine studies utilized computers as the primary hardware for their VR tasks (Cushman et al., 2008; Morganti et al., 2013; Lee et al., 2014; Kempe et al., 2015; Serino et al., 2015; Mohammadi et al., 2018; Zhang et al., 2021; Hogan et al., 2023; Rekers and Finke, 2023).

Four studies employed tablets (Zygouris et al., 2015; Eraslan Boz et al., 2020; Limoncu et al., 2021; Turner et al., 2021).

### 3.4 Computer-generated versus real scenarios

Out of the studies currently under examination, only three have utilized authentic scenarios as part of their research methodology (Cushman et al., 2008; Bruni et al., 2022; Pieri et al., 2022).

## 4 Discussion

The findings of this systematic review highlight a significant observation in the field of VR tools used for assessing memory. The primary focus of this review is the comprehensive collection of evidence that targeted three specific area of memory: episodic, prospective, and spatial memory.

The evaluation of episodic memory in VR settings has received significant attention and has become a subject of research interest. The emphasis on this aspect is not unexpected, given that VR technologies offer a distinctive medium for engaging participants in immersive and varied contexts, which can effectively assess their capacity to remember particular events, locations, and experiences. Scholars have utilized the immersive characteristics of VR in order to develop comprehensive episodic memory evaluations that closely resemble authentic real-world scenarios (Smith, 2019). Indeed, the studies being examined have purposefully endeavored to anchor their memory evaluation tasks within ecologically significant contexts. For example, certain studies have conducted their evaluations in familiar settings, including residential dwellings (Arvind Pala et al., 2014; Sauzéon et al., 2016), shops (Ouellet et al., 2018; Corriveau Lecavalier et al., 2020), and even simulated moving scenarios (Bruni et al., 2022). The utilization of these ecological settings has afforded participants with scenarios that closely approximate real-world circumstances. This exercise aimed to replicate a real-life memory challenge that individuals frequently face in their day-to-day experiences. The supermarket has been a commonly utilized ecological setting in numerous studies (Zygouris et al., 2015; Kempe et al., 2015; Eraslan Boz et al., 2020; Limoncu et al., 2021; Turner et al., 2021). The utilization of this particular context corresponds to the everyday encounters of numerous individuals and provides a practical framework for evaluating episodic memory in diverse shopping-related situations. Plancher et al. (2010) adopted an alternative methodology wherein participants were exposed to a simulated car accident scenario, followed by an assessment that tested their ability to recollect specific details and events associated with the high-stress situation. In addition, the researchers Gottlieb et al. (2022) conducted a virtual replication of the widely recognized Rey Auditory Verbal Learning Test (RAVLT). This study highlights the capacity of VR to imitate conventional neuropsychological memory assessments in settings that are both more regulated and closely aligned with real-world conditions.

The investigation of prospective memory, which pertains to the ability to recall and perform intended actions or tasks at a later time, has gained considerable attention in the realm of VR memory assessments. The inherent flexibility of VR environments enables the effective incorporation of prospective memory tasks within immersive settings. In such scenarios, individuals are tasked with the recollection and execution of intentions while actively engaging with and exploring the virtual realm (Lecouvey et al., 2019; Hogan et al., 2023). The emphasis on prospective memory highlights the capacity of VR tools to replicate and evaluate memory difficulties encountered in real-life scenarios.

The assessment of spatial memory has also been a significant area of focus in VR studies. The ability of virtual reality to accurately reproduce and manipulate spatial environments has facilitated the development of tasks that require individuals to navigate and remember intricate spatial layouts, which are integral to cognitive processes. The utilization of VR by researchers has enabled the immersion of individuals in various spatial contexts, thereby creating an advantageous environment for the examination of spatial memory processes.

Although the focus on these three memory domains is evident, it is crucial to acknowledge that the versatility of VR presents opportunities for evaluating a broader spectrum of memory functions. Potential future research endeavors may involve the investigation of additional subtypes of memory or the extension of the application of VR in the evaluation of less commonly examined memory domains such as semantic and autobiographical. The aforementioned findings indicate the necessity for a more extensive investigation into the various applications of VR in the assessment of memory. This exploration should take into account the potential contributions that this technology can make to the broader field of cognitive sciences and its practical implications in clinical practice.

Furthermore, another aim of this research was to evaluate the extent of convergence and divergence that can be observed when comparing memory assessments based on VR with conventional neuropsychological tests. Our analysis revealed a prominent pattern, indicating significant similarity between VR memory assessments and conventional neuropsychological tests. Specifically, VR memory tasks demonstrated notable and robust associations with memory assessments conducted using traditional methods. This implies that VR tasks have demonstrated the ability to capture memory processes related to episodic, prospective, and spatial memory, which are consistent with established neuropsychological assessments. The performance of participants in VR environments exhibited a strong correlation with their performance on established episodic memory assessments, highlighting the alignment in the memory processes being evaluated. In particular, regarding episodic memory, the results highlight the strong convergent validity of these virtual tools in the evaluation of episodic memory.

In the realm of prospective memory, the results of the study of Hogan et al. (2023) indicate that VR tasks are successful in capturing and reflecting the PM abilities that are evaluated by conventional measures. This finding provides evidence in favor of the validity of VR tasks as a means of assessing PM. Moreover, the study unveiled significant correlations between time-based prospective memory tasks conducted in VRPMST and the corresponding time- and event-based PM tasks conducted in laboratory-based dual-task paradigms. Furthermore, a significant and strong association was observed between both time and event-based PM as evaluated using VRPMST and the outcomes obtained from the Trail Making Test (TMT), the Hopkins Verbal Learning Test-Revised (HVLT-R), and the Montreal Cognitive Assessment (MoCA). The aforementioned correlations provide additional evidence supporting the notion that VRPMST encompasses both time-based and event-based aspects of prospective memory. These aspects have been found to have significant associations with established neuropsychological tests, thus highlighting the versatility and reliability of VRPMST as a tool for assessing PM.

The second study (Lecouvey et al., 2019) examined individuals diagnosed with AD and explored the correlation between prospective memory intentions and different cognitive functions. The results of this study provided valuable insights into this relationship: certain elements of intentions related to prospective memory are linked to different cognitive domains. The relevance of the correlation with TMT Part B is noteworthy due to its frequent utilization as an assessment tool for cognitive flexibility. Moreover, the observed association between TMT Part B and PM in individuals with AD underscores the interconnectedness between prospective memory and executive functions within this specific population.

The existing body of research investigating the use of virtual reality tests for spatial memory consistently demonstrates positive associations with conventional tests that evaluate memory and visuospatial capabilities. The consistent and strong correlation observed in these findings highlights the concurrent validity of the virtual reality tests in evaluating spatial memory and other cognitive functions that are interconnected.

The research conducted by Mohammadi et al. (2018) revealed noteworthy associations between their spatial memory task and established memory assessments, such as Rey immediate recall and delayed recall. This implies that the virtual reality task employed successfully captures and evaluates aspects of episodic memory, highlighting its efficacy as a dependable instrument for assessing memory functions within a realistic spatial framework.

Significant correlations were observed between the VR-MT and VR-MTM tests and Corsi’s supra-span task, which is a commonly employed assessment tool for evaluating visuospatial memory and navigation abilities. The results of this study highlight the correlation between the virtual reality assessments and well-established visuospatial tasks, thereby strengthening their concurrent validity.

There was a notable association observed between the Vienna task and visuospatial short-term memory. Nevertheless, the present task failed to exhibit a substantial correlation with episodic memory, as assessed by the proportion of elements recalled during the delayed free recall phase of the Rey-Osterrieth Complex Figure Test (ROCFT). The particularity of this task underscores its capacity to evaluate specific visuospatial memory domains, without necessarily encompassing episodic memory.

In contrast, the study conducted by Mohammadi et al. (2018) found a correlation between the ROCFT and simplified Rey figure test (SRFT) with the virtual neighborhood task. Similarly, Lee et al. (2014) observed a correlation between ROCFT and SRFT with the virtual radial arm maze (VRAM). Additionally, Tippett et al. (2009) proposed a task that also exhibited a correlation with ROCFT and SRFT. These observations imply that the conventional visuospatial memory tests exhibit similar characteristics to their virtual reality counterparts, thus indicating their concurrent validity in evaluating spatial memory.

In conclusion, the findings presented in this study underscore the strong concurrent validity of virtual reality assessments in measuring spatial memory. These assessments consistently demonstrate significant correlations with conventional measures used to evaluate memory, visuospatial skills, executive functions, and overall cognitive functioning. This highlights the potential of utilizing virtual spatial memory assessments as valuable instruments for evaluating diverse cognitive functions within authentic spatial contexts.

Nevertheless, our analysis also uncovered fascinating occurrences of disparity between VR based memory evaluations and traditional neuropsychological examinations. The divergence that stood out the most was observed in the study conducted by Plancher et al., where a virtual task was found to lack correlations with conventional memory assessments. This discovery implies that the particular virtual reality task in question may engage distinct memory components or cognitive mechanisms that are not fully assessed by traditional assessments. Additionally, it is important to highlight that certain novel VR tools demonstrated associations with functions that extend beyond memory. Several studies have demonstrated associations between VR tasks and cognitive functions such as memory and executive functions. Notable examples include the works of Arvind Pala et al. (2014), Zygouris et al. (2015), Sauzéon et al. (2016), Ouellet et al. (2018), Eraslan Boz et al. (2020), Limoncu et al. (2021), Bruni et al. (2022), and Gottlieb et al. (2022). This implies that the utilization of VR memory tasks can be advantageous in the simultaneous evaluation and differentiation of memory and executive function elements, thereby offering a more comprehensive understanding of cognitive profiles.

In addition, a number of the VR assessments that were examined displayed significant correlations with overall cognitive functioning, suggesting that they have the potential to be useful instruments for evaluating an individual’s overall cognitive status.

The findings of this study highlight the potential of VR based memory assessments as comprehensive instruments for evaluating the overall cognitive wellbeing of individuals.

The findings of our review are noteworthy as they establish a connection between VR memory assessments and executive functions, as well as general cognitive functions. These correlations serve as compelling evidence for the effectiveness of the functional approach utilized in these assessments. The aforementioned result highlights the potential of memory tasks based on VR to provide a comprehensive and integrated understanding of cognitive function, in contrast to the conventional approach driven by specific constructs.

The correlations observed concerning executive functions indicate that virtual reality memory assessments effectively engage memory processes in a manner that is inherently interconnected with other cognitive domains. This integration is consistent with the principles of functional assessment, which posits that cognitive functions are not discrete constructs but rather function in conjunction with one another within the larger cognitive system. Virtual reality has emerged as a promising method for evaluating the relationship between memory and executive functions. By utilizing VR-based memory tasks, researchers can gain a more comprehensive and realistic understanding of cognitive performance, thereby enhancing the ecological validity of their assessments.

These correlations highlight the notion that VR memory assessments do not adhere to a strict construct-driven methodology, but instead adhere to a functional approach that aims to measure cognitive processes as they occur organically within intricate, real-world situations. The functional perspective is highly appropriate for comprehensively understanding the complex interaction among memory, executive functions, and overall cognitive performance, thereby offering a more comprehensive assessment of cognitive wellbeing. Therefore, the utilization of VR as a tool for evaluating memory holds great potential for advancing cognitive research and clinical applications, as it allows for a more comprehensive and realistic understanding of cognitive abilities.

Another aspect explored in this review pertained to the discernment of whether the settings employed for memory evaluations were computer-generated or obtained through the utilization of 360-degree technologies. The objective of this investigation was to analyze the technological underpinnings of the virtual scenarios utilized in the research, providing insight into the adaptability and benefits associated with various methods of constructing immersive environments for cognitive evaluations. Out of the studies currently under examination, only three have utilized authentic scenarios as part of their research methodology (Cushman et al., 2008; Bruni et al., 2022; Pieri et al., 2022). Overall, the decision between computer-generated virtual environments and real scenarios captured with 360° technologies in memory assessment tasks necessitates a compromise between the level of control exerted over the environment and the degree of realism achieved. The selection of a particular approach should be in accordance with the research objectives, taking into account the inherent trade-offs between these crucial dimensions. In situations where ecological validity is of utmost importance, the utilization of authentic scenarios may be more favorable. However, for assessments that require strict control and standardization, computer-generated environments present distinct advantages in terms of customization and reproducibility. Researchers should carefully consider these factors in relation to their research objectives, the specific population being studied, and the resources at their disposal.

One noteworthy constraint of this systematic review is the limited accessibility of the virtual reality memory assessments utilized in the studies under review. The review sought to comprehensively synthesize the extant literature; however, it was noted that only one study made their VR tasks openly accessible and downloadable (Rekers and Finke, 2023). The restricted accessibility of these studies poses a challenge to the wider scientific community in terms of replicating, validating, and expanding upon their findings. This emphasizes the importance of promoting transparency and adopting open science practices within the realm of VR-based memory assessment. Furthermore, the studies included in the analysis demonstrated significant variability in the VR hardware and software utilized for evaluating memory performance. The range of variability encompassed various technologies, including video projectors, head-mounted displays, computer interactions, and tablets. The presence of diverse hardware and software options in VR can pose challenges when attempting to compare and integrate research findings. This is because the selection of specific hardware and software can have a substantial influence on the user’s experience and the outcomes of VR tasks. The presence of heterogeneity underscores the importance of implementing standardization within the discipline.

## Author contributions

VM: Conceptualization, Data curation, Formal analysis, Investigation, Methodology, Project administration, Software, Supervision, Validation, Visualization, Writing – original draft, Writing – review & editing. ES: Writing – review & editing. FB: Writing – review & editing. SA: Writing – review & editing. SD: Writing – review & editing. MC: Writing – review & editing. PC: Methodology, Writing – review & editing. EP: Conceptualization, Writing – review & editing.
